# Inverse Vulcanization of Styrylethyltrimethoxysilane–Coated Surfaces, Particles, and Crosslinked Materials

**DOI:** 10.1002/anie.202006522

**Published:** 2020-10-05

**Authors:** Johannes M. Scheiger, Chatrawee Direksilp, Patricia Falkenstein, Alexander Welle, Meike Koenig, Stefan Heissler, Jörg Matysik, Pavel A. Levkin, Patrick Theato

**Affiliations:** ^1^ Institute of Chemical Technology and Polymer Chemistry Karlsruhe Institute of Technology (KIT) Engesserstrasse 20 76131 Karlsruhe Germany; ^2^ Institute of Biological and Chemical Systems—Functional Materials Systems (IBCS-FMS) Karlsruhe Institute of Technology (KIT) Hermann-von-Helmholtz-Platz 1 76344 Eggenstein-Leopoldshafen Germany; ^3^ The Petroleum and Petrochemical College (PPC) Chulalongkorn University Soi Chulalongkorn 12, Phayathai road, Pathumwan Bangkok 10330 Thailand; ^4^ Institute of Analytical Chemistry Leipzig University Linnéstrasse 3 04103 Leipzig Germany; ^5^ Institute of Functional Interfaces (IFG) Karlsruhe Institute of Technology (Campus North) Hermann-von-Helmholtz-Platz 1 76344 Eggenstein-Leopoldshafen Germany; ^6^ Karlsruhe Nano Micro Facility (KNMF) Karlsruhe Institute of Technology (Campus North) Germany; ^7^ Soft Matter Synthesis Laboratory Institute for Biological Interfaces III Karlsruhe Institute of Technology (KIT) Hermann-von-Helmholtz-Platz 1 76344 Eggenstein-Leopoldshafen Germany

**Keywords:** alkoxy silanes, coatings, inverse vulcanization, mercury remediation, sulfur

## Abstract

Sulfur as a side product of natural gas and oil refining is an underused resource. Converting landfilled sulfur waste into materials merges the ecological imperative of resource efficiency with economic considerations. A strategy to convert sulfur into polymeric materials is the inverse vulcanization reaction of sulfur with alkenes. However, the materials formed are of limited applicability, because they need to be cured at high temperatures (>130 °C) for many hours. Herein, we report the reaction of elemental sulfur with styrylethyltrimethoxysilane. Marrying the inverse vulcanization and silane chemistry yielded high sulfur content polysilanes, which could be cured via room temperature polycondensation to obtain coated surfaces, particles, and crosslinked materials. The polycondensation was triggered by hydrolysis of poly(sulfur‐*r*‐styrylethyltrimethoxysilane) (poly(S_n_‐*r*‐StyTMS) under mild conditions (HCl, pH 4). For the first time, an inverse vulcanization polymer could be conveniently coated and mildly cured via post‐polycondensation. Silica microparticles coated with the high sulfur content polymer could improve their Hg^2+^ ion remediation capability.

## Graphical abstract

Marrying inverse vulcanization and silane chemistry yields solution‐processable high sulfur content polysulfide‐alkoxysilanes, which could be cured via polycondensation. With this strategy, coated surfaces and particles as well as crosslinked materials can be obtained. This method extends the applicability and control of materials prepared via inverse vulcanization.

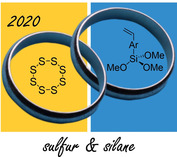

## Introduction

The inverse vulcanization allowed repurposing of sulfur, a multi‐million‐ton side product of oil and natural gas refining, to form polymeric materials.^1^, ^2^, ^3^, ^4^, ^5^ These materials have been shown to be inexpensive and useful in applications such as infrared optics,^6^, ^7^, ^8^, ^9^, ^10^, ^11^, ^12^, ^13^ catalysis,^14^ Li‐sulfur batteries,^15^, ^16^ pollutant remediation,^17^, ^18^, ^19^, ^20^, ^21^ antibacterial surfaces,^22^, ^23^ templates,^24^ healable materials,^25^, ^26^ fertilizers,^27^, ^28^ adhesives,^29^ or as insulators.^30^, ^31^ With the aid of catalysts or the so‐called dynamic covalent polymerization, the monomer (i.e. crosslinker) pool could be expanded beyond highly reactive styrene or norbornene type double bonds to vinyl siloxanes, methacrylates, or allyl ethers.^32^, ^33^, ^34^, ^35^, ^36^ Apart from design principles abbreviated by hasell and chalker to alter thermomechanical properties such as the glass transition temperature *T*_G_ or the compression modulus by varying the type and stoichiometry of the comonomers, the inverse vulcanization lacks control over basic polymer parameters such as the solubility or the molecular weight.^37^, ^38^ Typically, materials are formed by reacting elemental sulfur with a crosslinker, with the decisive parameter being the stoichiometry. Resulting polymers are either oily liquids, soluble solids or highly crosslinked, insoluble materials, depending on their sulfur content. No strategy of a solution processable polysulfide which can be mildly cured into an insoluble thermoset had been introduced yet. We hypothesized, that silane chemistry could be a potent tool to form high‐sulfur content materials with mild post‐polymerization chemistry. Silanes are a compound class of large relevance, with applications ranging from academic research to industrial applications. They are the most important chemical tool to covalently functionalize ceramics, glasses, or metals to modify or protect their surface. As binder between organic and inorganic moieties, they are particularly relevant for applications such as membranes, filters or optics where the surface or interface properties are decisive for the functionality of a material. Silanes typically consist of organic moieties and up to three chloro‐ or alkoxy substituents bound to a silicon atom. The chloro‐ or alkoxy substituents can be hydrolyzed to form silanol bonds, which enable a polycondensation or the attachment to various surfaces. When the polycondensation is performed in bulk, trichloro‐ or trialkoxysilanes yield insoluble crosslinked polysiloxanes. It was assumed, that the alkoxy functionality of trimethoxysilanes would be preserved during an inverse vulcanization reaction and thus would provide access to high sulfur content polysiloxanes after a post‐polycondensation reaction. Styrylethyltrimethoxysilane (StyTMS) was selected since styrene is known to undergo inverse vulcanization reactions.^15^ Herein, we report the inverse vulcanization reaction of sulfur with styrylethyltrimethoxysilane and the follow‐up siloxane chemistry of the formed poly(S_*n*_‐*r*‐StyTMS), which could be polycondensated using a mild chemical trigger (HCl, pH 4) to yield coated surfaces, particles, and crosslinked networks with a high sulfur content. Various substrates such as surfaces, filters and particles could be coated and control over thickness is demonstrated. Coated silica particles showed an increase in their Hg^2+^ remediation capability. Obtained materials were investigated with surface sensitive methods such as AFM, ATR FTIR, EDX, ellipsometry, ToF SIMS and XPS, as well as bulk methods such as solid‐state NMR, TGA‐MS, DSC, and PXRD.

## Results and Discussion

**Scheme 1:**
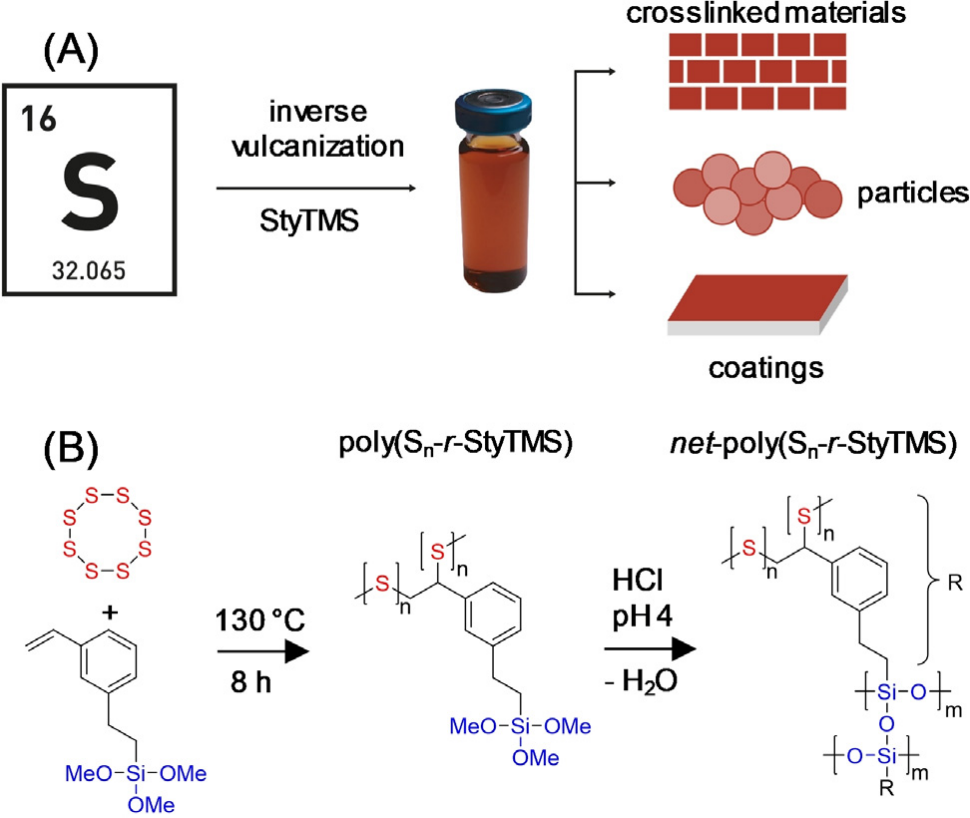
A) Conversion of sulfur with styrylethyltrimethoxysilane (StyTMS) into a soluble poly(S_*n*_‐*r*‐StyTMS) building block. The three methoxy functional groups can be hydrolyzed and polycondensated to yield high sulfur content coatings on surfaces and particles as well as crosslinked bulk *net*‐poly(S_*n*_‐*r*‐StyTMS). B) Reaction scheme of the inverse vulcanization of styrylethyltrimethoxysilane (StyTMS) with elemental sulfur and the subsequent hydrolysis and polycondensation to yield *net*‐poly(S_*n*_‐*r*‐StyTMS).

Styrylethyltrimethoxysilane (StyTMS) was reacted with elemental sulfur in an inverse vulcanization reaction in bulk at 130 °C for 8 h and the product of the reaction, poly(S_*n*_‐*r*‐StyTMS) could undergo hydrolysis and polycondensation to produce coated surfaces and particles as well as crosslinked materials with a maximum sulfur content of 35 wt % (Scheme 1). Upon reaction of StyTMS with elemental sulfur, C=C double bonds were consumed quantitatively, while the alkoxy functionalities remained unchanged as confirmed via ^1^H NMR spectroscopy (Figure S1). The product of the inverse vulcanization reaction, poly(S_*n*_‐*r*‐StyTMS), was of low molecular weight (M_W_ 1300 g mol^−1^) and well soluble in THF (Figure S2).

Addition of 5 vol% of dilute HCl (pH 4) to a solution of poly(S_*n*_‐*r*‐StyTMS) in THF caused hydrolysis of the methoxy groups, which initiated a polycondensation reaction. As a result, the molecular weight of polymers in solution increased to M_W_ 5500 g mol^−1^ (Figure S3). Removal of volatiles accelerated the polycondensation as indicated by the formation of an orange‐red powder which was insoluble in THF, toluene, hexanes, and CS_2_ because of Si‐O‐Si crosslinking. This was observed for all weight ratios of sulfur tested (17–35 wt % by elemental analysis), which is different from most reports, where insolubility typically occurred for high sulfur contents.^15^ The heat capacity of these materials decreased mildly with increasing sulfur content (Figure S4). The highest sulfur content in crosslinked *net*‐poly(S_*n*_‐*r*‐StyTMS) was determined to be 35 wt % by elemental analysis, when a 1:1 weight ratio of sulfur and StyTMS was used. This is below the highest value reported for materials made via inverse vulcanization (90 wt % sulfur) but still remarkable for a polymeric material.^15^ Considering that there is a large share of ethylphenethyltrimethoxysilane impurity (ca. 27 % as determined via ^1^H‐NMR spectroscopy) in commercial StyTMS which does not contribute to sulfur fixation (Figure S1). Thus, the weight ratio of bound sulfur could be increased to ca. 50 % using purer StyTMS. The stability of *net*‐poly(S_*n*_‐*r*‐StyTMS) against depolymerization into sulfur was confirmed with differential scanning calorimetry (DSC) and powder *x*‐ray diffractometry (PXRD) of powders aged for two months, whereas thermogravimetric analysis (TGA) coupled mass spectrometry showed the release of SO_2_ and CO_2_ upon thermal degradation of *net*‐poly(S_*n*_‐*r*‐StyTMS) above 220 °C (Figure S5).

**Figure 1:**
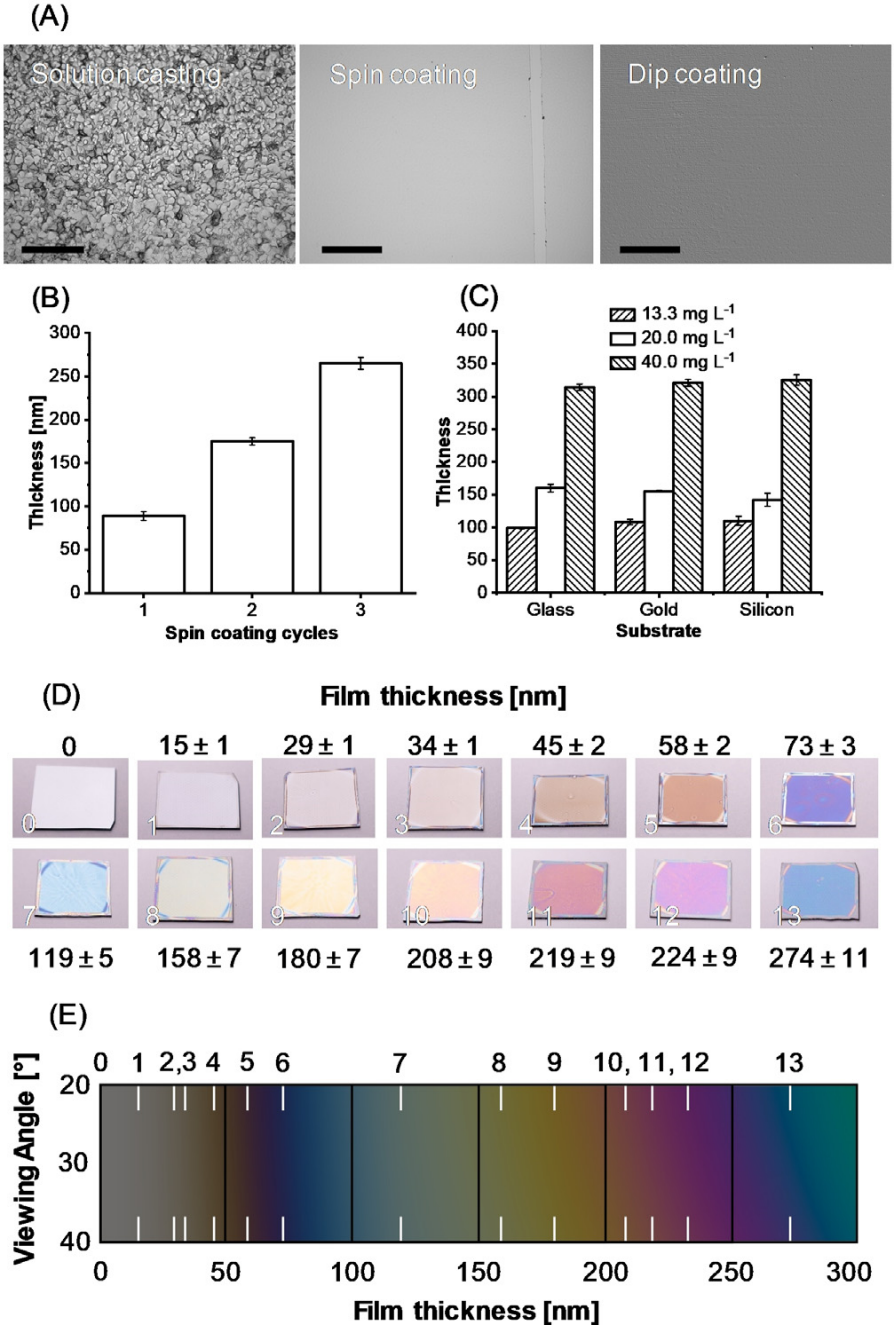
A) Microscopy images of glass surfaces coated with *net*‐poly(S_*n*_‐*r*‐StyTMS) with different coating techniques. The spin‐coated surface was scratched with a needle to visualize the coating. An “Emboss (strong)” filter was used for the dip coated sample, to enhance the visibility of the micropores on the surface. Scalebars are 200 μm. B) Influence of the number of spin‐coating cycles on the film thickness on glass as determined with atomic force microscopy. C) Influence of the poly(S_*n*_‐*r*‐StyTMS) concentration on the film thickness on different substrates via spin coating (glass, silicon, gold). D) Digital images of silicon wafers spin coated with *net*‐poly(S_*n*_‐*r*‐StyTMS) taken at a camera viewing angle of approximately 30° (relative to the surface normal). The different colors are caused by thin film interference. By varying the coating thickness on the nanoscale, different surface colors were obtained. E) Theoretical calculation of thin film interference colors in dependence of the film thickness and viewing angle. Parameters required for the calculation were based on the Cauchy model with absorbance corrections. Samples are indicated as white stripes. Calculation parameters and details are summarized in the Supporting Information.

In order to explore future applications of sulfur containing polymers, strategies to bind high sulfur content materials to surfaces are required and relevant. For this, poly(S_*n*_‐*r*‐StyTMS) was dissolved in THF and was hydrolyzed with 5 vol % of dilute HCl (pH 4). This solution was then employed for different coating techniques and glass, silicon, and gold substrates were coated via spin coating, dip coating, and solution casting (Figure 1 A). The obtained topologies differed strongly for the different coating techniques and concentrations used. Polymerization induced phase separation was observed in solution and is thus believed to influence the surface topology (Figure S3). Solution casting led to a micro‐rough surface topology. For dip coating, the topology depended strongly on the speed of substrate removal from the silanization solution (Figure S6). Substrates were immersed for 5 min and were then removed with a defined speed of 40, 45, 125, and 760 mm min^−1^ using a dip‐coater. Dip‐coated surfaces showed elongated features with widths of several micrometers and heights increasing from 24 to 70 nm with increasing substrate removal speed. Spin‐coating on glass yielded homogenous micro‐ and nano‐porous films depending on the concentration of the poly(S_*n*_‐*r*‐StyTMS) solution and amount of spin‐coating cycles (Figure S7). Briefly, high concentrations of poly(S_*n*_‐*r*‐StyTMS) led to the formation of micropores (ca. 6–7 μm), presumably from polymerization induced self‐assembly and THF evaporation. The pore sizes reduced (<1 μm) for repeated spin‐coating cycles and for lower concentrations of poly(S_*n*_‐*r*‐StyTMS) (Figure S8). To control the film thickness, the hydrolyzed poly(S_*n*_‐*r*‐StyTMS) solution was spin‐coated for different numbers of spin‐coating cycles and with varying concentrations of hydrolyzed poly(S_*n*_‐*r*‐StyTMS) and was investigated with AFM scratch analysis (Figure S9). For one, two, and three spin‐coating cycles the height of *net*‐poly(S_*n*_‐*r*‐StyTMS) on glass increased almost linearly from 90 to 175 nm to 265 nm (Figure 1 B). By reducing the concentration of poly(S_*n*_‐*r*‐StyTMS) from 40 over 20 to 13.3 mg mL^−1^ (referred to the initial amount of StyTMS) in THF, the film thicknesses obtained after spin‐coating on different substrates were between 314–325 nm, 142–160 nm, and 99–110 nm, respectively. The substrates thus did not seem to influence the film thickness significantly (Figure 1 C). A series of silicon wafers coated with different thicknesses were prepared using spin‐coating and different concentrations of poly(S_*n*_‐*r*‐StyTMS). Due to the thin‐film interference effect on the reflective silicon surfaces, the difference in film heights could be observed with bare eyes (Figure 1 D). The surfaces were investigated with ellipsometry and the observed interference colors were compared with a calculated thin film interference pattern for validation (Figure 1 E, Figure S10). The close match between calculated and obtained interference colors proved that the color of *net*‐poly(S_*n*_‐*r*‐StyTMS)surfaces could be accurately predicted and predetermined. Hence, the disadvantageous intrinsic, red coloration of polysulfides could be overcome by exploiting thin film interference, which could be of interest for applications in optical coatings (e.g. effect lacquers).

**Figure 2:**
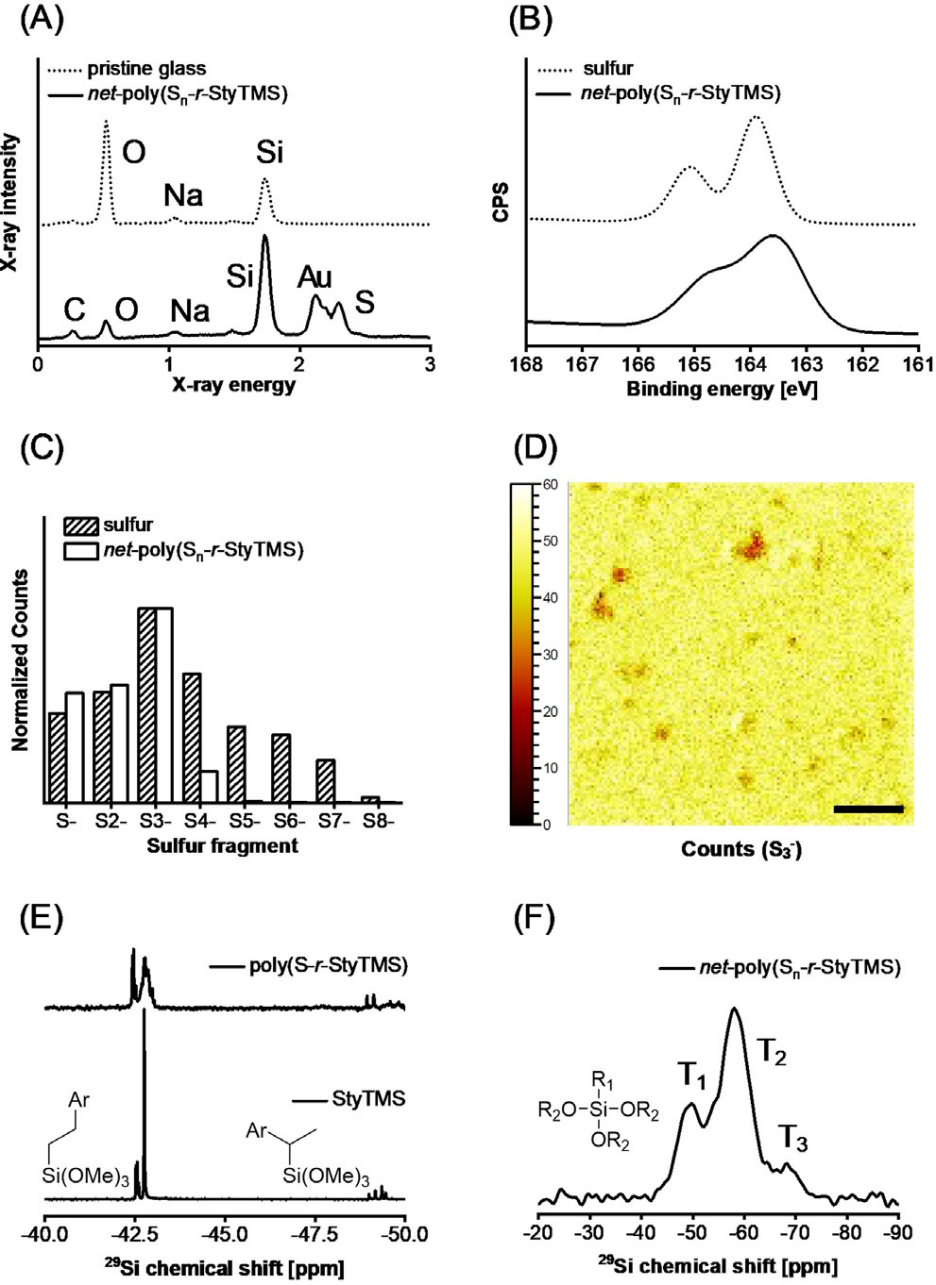
A) EDX spectra of glass with and without a *net*‐poly(S_*n*_‐*r*‐StyTMS) coating. Characteristic carbon and sulfur peaks can be observed for the coated sample. B) XPS spectra of elemental sulfur and *net*‐poly(S_*n*_‐*r*‐StyTMS). While elemental sulfur showed a clear doublet due to spin‐orbit coupling, the polysulfur coating showed a broader energy distribution arising from multiple, subtly different sulfur species. The energy distribution of the coating shifted towards higher energies, with the maximum at 164.6 eV being typical for sulfur bound to carbon. C) ToF‐SIMS analysis of the *net*‐poly(S_*n*_‐*r*‐StyTMS) coating. Relative distribution analysis of polysulfide fragments (S_*n*_^−^) for elemental sulfur and the *net*‐poly(S_*n*_‐*r*‐StyTMS) coating. D) S_3_^−^ fragment mapping via ToF‐SIMS showing a homogenous distribution of trisulfide in the coating within the examined area of 500×500 μm (0.25 mm^2^). E) ^29^Si NMR spectra of StyTMS (bottom) and poly(S_*n*_‐*r*‐StyTMS) (top) in solution (CDCl_3_) at identical expansion. The eight peaks in StyTMS correspond to 1‐ and 2‐silyl constitution isomers and the respective *m*‐ and *p*‐isomers of the two aryl groups (Ar=styryl or ethylphenyl). After the inverse vulcanization, the four peaks corresponding to StyTMS isomers broadened and decreased in intensity, whereas the respective ethylphenethyltrimethoxysilane isomers remained unchanged. F) ^29^Si CP‐MAS NMR spectrum of powdered *net*‐poly(S_*n*_‐*r*‐StyTMS) showing the T_1_, T_2_, and T_3_ Si species after the polycondensation. The majority of silicon atoms was found to be in a T_2_ ((SiR_1_(OR_2_)O)_*n*_) binding state. R_1_ can be styrylethyl or ethylphenethyl. R_2_ can be ‐H, ‐Me, or ‐Si. The relative amount of T_1_, T_2_, and T_3_ was determined via line fitting integration of a HPDEC spectrum to be 4.5, 10.6, and 1.0, respectively.

The *net*‐poly(S_*n*_‐*r*‐StyTMS) coated surfaces were analyzed with energy dispersive *x*‐ray spectroscopy (EDX), *x*‐ray photoelectron spectroscopy (XPS) and time‐of‐flight secondary ion mass spectrometry (ToF SIMS). All three methods conclusively and independently proved the presence of sulfur on the coated surfaces. Prior to measurements, surfaces were washed with excess CS_2_ and THF to remove traces of elemental sulfur (Figure 2). EDX confirmed the presence of the characteristic elements, sulfur and silicon, on the coated surfaces (Figure 2 A), while XPS showed various sulfur‐carbon binding states (Figure 2 B). Elemental sulfur powder was used as a reference for XPS. The obtained peak maximum energy of 164.1 eV was referenced to the literature value of 163.9 eV and the same shift (−0.2 eV) was applied to the obtained XPS spectra of the *net*‐poly(S_*n*_‐*r*‐StyTMS) coated sample. In the spectrum of elemental sulfur, the 2p‐electron energy levels were distinctly split up into a doublet. For the *net*‐poly(S_*n*_‐*r*‐StyTMS) coated sample a broadening of the energy distribution was observed. This is due to the presence of sulfur in many different but similar binding states, that is, C‐S_*n*_‐C with n=ℕ>0 . An energy distribution shift to higher binding energies from 163.9 eV (sulfur) to 164.6 eV *net*‐poly(S_*n*_‐*r*‐StyTMS) was observed, which indicated the presence of C−S bonds. Attempts to gain an improved understanding of the S‐S species with Raman spectroscopy failed due to sample fluorescence, whereas ATR FTIR (attenuated total reflection Fourier transform infrared spectroscopy) could only confirm the presence of the organic and siloxane moieties (Figure S11). Thus, the ToF‐SIMS (time‐of‐flight secondary ion mass spectrometry) method was employed to confirm the presence of polysulfur chains on the surface (Figure 2 C). The distribution of sulfur associated ions (i.e. S_*n*_^−^) was homogenous within the examined surface area (Figure 2 D, Figure S12 and S13). ToF‐SIMS experiments with elemental sulfur powder were conducted under the same ionization conditions to gain insight into the fragmentation patterns of sulfur‐sulfur bonds. For elemental sulfur, fragments up to S_8_^−^ were detected, while for the *net*‐poly(S_*n*_‐*r*‐StyTMS) no polysulfide fragments heavier than S_4_^−^ were traced. The most dominant fragment was S_3_^−^ for both elemental sulfur and the *net*‐poly(S_*n*_‐*r*‐StyTMS) surfaces. Thus, it could be concluded that the fragmentation pattern did not represent the abundance of the sulfur species. The obtained mass distribution in the *net*‐poly(S_*n*_‐*r*‐StyTMS) coating indicated that the sulfur chains were shorter than S_8_ and presumably between S_1_‐S_5_. Due to the high C−S bond energy of ca. 370 kJ mol^−1^ it could be reasoned that central S−S bonds or α‐C−S bonds break more readily upon ion bombardment (S−S bond energy in disulfides and cyclooctasulfur is ca. 270 kJ mol^−1^ and 174 kJ mol^−1^, respectively).^39^, ^40^ Such an effect would cause a decrease in the observable length of the polysulfide fragments.

To investigate the inverse vulcanization and polycondensation process from the perspective of silicon atoms, ^29^Si NMR was conducted in solution and solid state (Figure 2 E and F). For the starting material, eight different silicon species were found, which corresponded to the 1‐, and 2‐silyl constitution isomers of *m*‐, *p*‐StyTMS and the ethylphenethyltrimethoxysilane impurity. Upon reaction with sulfur, peaks associated to StyTMS isomers broadened strongly due to polymerization, whereas the ethyl isomers remained unchanged. The 1‐silyl *m*‐ and *p*‐isomers of StyTMS experienced a noticeable shift from −49.4 and −49.5 ppm to −49.6 and −51.1 ppm, respectively, presumably due to the closer distance to the aryl group compared to the 2‐silyl isomers. ^29^Si cross polarization (CP) magic‐angle spinning (MAS) NMR of the solid, powdered *net*‐poly(S_*n*_‐*r*‐StyTMS) revealed the relative distribution of silicon species T_1_, T_2_, and T_3_. Line fitting integration of both CP and high power decoupled (HPDEC) MAS NMR spectra (Figure S14) resulted in an average of 1.8 Si‐O‐Si bonds per silicon center, which deviates from a fully crosslinked siloxane (3.0 Si‐O‐Si bonds per silicon center) and is closer to a linear polysiloxane (2.0 Si‐O‐Si bonds per silicon center). Since only solids were obtained after polycondensation, it could be concluded that the contribution of sulfur to the crosslinking was essential.

**Figure 3:**
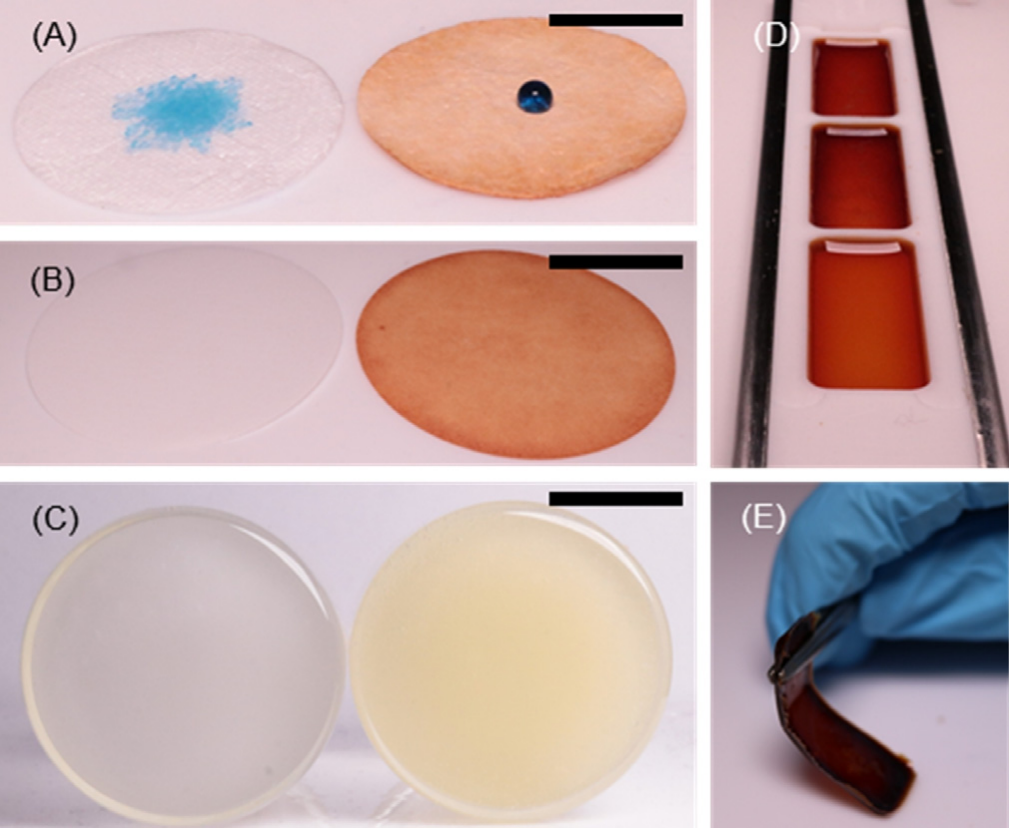
Digital images of a A) glass fiber filter, a B) cellulose filter, and C) silica gel before (left) and after (right) coating with *net*‐poly(S_*n*_‐*r*‐StyTMS). The hydrophobicity of the filter surfaces was increased after coating with *net*‐poly(S_*n*_‐*r*‐StyTMS) as seen from the formation of a water droplet (contains blue dye) instead of droplet spreading. Scalebars are 1, 3, and 1 cm, respectively. D) Digital images of solution casting of hydrolyzed poly(S_*n*_‐*r*‐StyTMS) from THF in a Teflon mold and E) the as prepared *net*‐poly(S_*n*_‐*r*‐StyTMS) film. Dimensions of the film are 15×37×1 mm (length×width×height).

With the developed method, *net*‐poly(S_*n*_‐*r*‐StyTMS) could be conveniently and homogenously coated onto various commonly used filter materials, such as cellulose, silica particles or glass fibers (Figure 3). Due to the low thickness of the coating, the fibrous surface structure of cellulose filters remained unchanged, whereas its presence could be readily detected with EDX (Figure S15). An inherent advantage of *net*‐poly(S_*n*_‐*r*‐StyTMS) for filter applications over previously reported polysulfides was the insolubility in solvent such as THF, hexanes, toluene, and CS_2_ and thus compatibility with organic solvents for practical applications. Solution casting of hydrolyzed poly(S_*n*_‐*r*‐StyTMS) in a Teflon mold yielded plastic films. Upon drying the films at room temperature overnight, they became brittle, which indicated a completion of the crosslinking via polycondensation.

**Figure 4:**
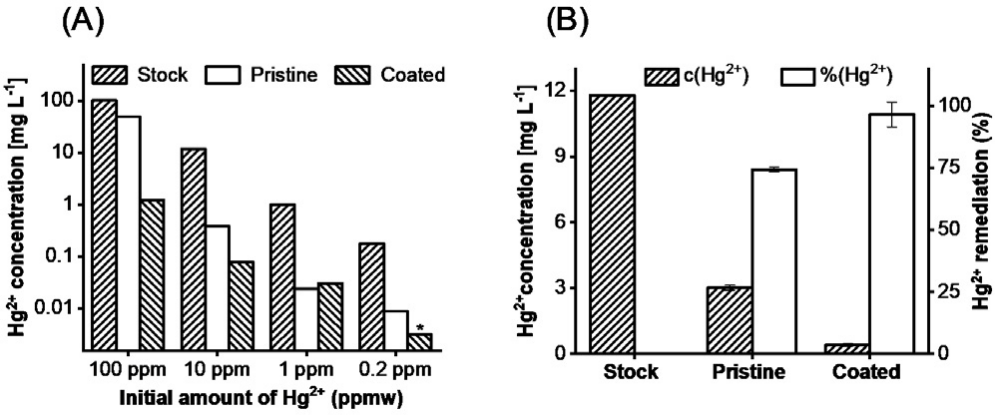
A) Mercury remediation experiments evaluated with hydride‐AAS for different initial concentrations of Hg^2+^. The difference in remediation performance between pristine and *net*‐poly(S_*n*_‐*r*‐StyTMS) coated silica microparticles increased with increasing initial Hg^2+^ concentration. B) Mercury remediation experiments for an initial Hg^2+^ concentration of approximately 10 ppmw. A decrease of Hg^2+^ concentration from 11.79 mg L^−1^ to 0.41 mg L^−1^ was observed, whereas the pristine silica control could decrease the Hg^2+^ concentration to 3.02 mg L^−1^. Values are given as average of three measurements with the error bars being the standard deviation.

To demonstrate the use of filter materials coated with a high sulfur content polymer, we functionalized silica microparticles with *net*‐poly(S_*n*_‐*r*‐StyTMS) and tested their change in mercury remediation properties. The successful surface modification was apparent by the color change from white pristine silica particles to pale yellow coated silica microparticles (Figure 3 C). DLS (dynamic light scattering) measurements of silica nanoparticle as a model system confirmed the successful modification of the silica particle surfaces. After immersing silica nanoparticles in hydrolyzed poly(S_*n*_‐*r*‐StyTMS) for 2 h at rt, their radius increased by Δ 30 nm (Figure S16). A similar coating process was originally published by the group of Hasell et al., who coated silica particles with a soluble limonene based polysulfide.^34^ To test the mercury uptake, 200 mg of *net*‐poly(S_n_‐*r*‐StyTMS) coated silica microparticles (40–63 μm) were stirred in 10 mL of HgCl_2_ (15 mg L^−1^) for one hour. The Hg^2+^ concentration in the solution was then determined by hydrid‐AAS (atom absorption spectroscopy) according to a reported method.^41^ As a control, pristine silica microparticles were used (Figure 4).

The concentration of Hg^2+^ decreased by 97 % from 11.79 mg L^−1^ to mg 0.41 mg L^−1^ after remediation with *net*‐poly(S_*n*_‐*r*‐StyTMS) coated silica microparticles, whereas the silica control could remove 74 % to a concentration of 3.02 mg L^−1^. The performance of particles coated with poly(S_*n*_‐*r*‐StyTMS) compared well to other materials prepared via inverse vulcanization reported previously.^19^, ^42^ Due to the polar siloxane and silanol groups, even the pristine silica microparticles could remediate Hg^2+^.^43^, ^44^ The difference between pristine silica microparticles and *net*‐poly(S_*n*_‐*r*‐StyTMS) coated silica microparticles became more pronounced for higher initial concentrations of Hg^2+^, which is in accordance with the observations of the group of Hasell (Figure 4 A). While *net*‐poly(S_*n*_‐*r*‐StyTMS) coated microparticles were able to remediate >97 % of Hg^2+^ cations throughout the tested Hg^2+^ concentration range, the remediation capability of pristine silica microparticles sharply decreased for an initial concentration of 100 mg L^−1^ HgCl_2_ (52 %). It should be noted that the content *net*‐poly(S_*n*_‐*r*‐StyTMS) as the functionality enhancing compound in the coated particles was low (<8 wt %), highlighting the value and efficiency of defined surface chemistries.

## Conclusion

The inverse vulcanization reaction of styrylethyltrimethoxysilane (StyTMS) with sulfur was reported. The obtained poly(S_*n*_‐*r*‐StyTMS) could undergo hydrolysis and polycondensation, which could be used to coat surfaces and particles. The sulfur content in *net*‐poly(S_*n*_‐*r*‐StyTMS) was 35 wt %, which is a remarkable amount for a solution processable and curable coating material. After curing, coatings and crosslinked materials were insoluble in organic solvents such as THF or CS_2_, extending the range of potential applications of polymers from inverse vulcanization. The thickness of spin‐coated films could be controlled and visualized. Due to the thin‐film interference effect, the coatings could appear in different colors (e.g. blue, purple, yellow) other than the typical red color of polysulfides. Silica particles coated with *net*‐poly(S_*n*_‐*r*‐StyTMS) improved their Hg^2+^ adsorption performance from 74 % to 97 % (*c*_0_=11.8 mg L^−1^). This work demonstrated the compatibility of silane chemistry with the inverse vulcanization, which opened new pathways for the mild post‐processing of high sulfur content coatings and bulk materials. Marrying silane chemistry and inverse vulcanization bears the potential to drive high sulfur content polymers closer to applications by offering controlled chemistries for crosslinking and coating. Relevant applications of high sulfur content polymers, such as antibacterial surfaces and mercury remediation, are surface controlled. Thus, the concepts presented herein can stimulate ideas for improved designs for such materials.

## Conflict of interest

The authors declare no conflict of interest.

## Supporting information

As a service to our authors and readers, this journal provides supporting information supplied by the authors. Such materials are peer reviewed and may be re‐organized for online delivery, but are not copy‐edited or typeset. Technical support issues arising from supporting information (other than missing files) should be addressed to the authors.

Supplementary
