# Supplementary material for: Inverse Vulcanization of Styrylethyltrimethoxysilane–Coated Surfaces, Particles, and Crosslinked Materials
Source: Angew Chem Int Ed Engl. 2020 Oct 5;59(42):18639–45. doi: 10.1002/anie.202006522 (PMC7589442; doi:10.1002/anie.202006522)
Supplement: Supplementary file 1 — Supplementary [file ANIE-59-18639-s001.pdf]

Table of content

Experimental Section 2-4
Methods 5-8
Figure S1: ^{1}H NMR 9-10
Figure S2 and S3: SEC 11
Figure S4: Heat capacity of *net*-poly(S_n-*r*-StyTMS) with different sulfur contents 12
Figure S5: PXRD, DSC and TGA MS 13-14
Figure S6: Light microscopy and AFM of dip-coated surfaces 15
Figure S7: Digital microscopy of spin-coated surfaces 16
Figure S8: AFM of spin-coated surfaces 17
Figure S9: AFM scratch analysis of spin-coated surfaces 18
Figure S10: Ellipsometry 19
Figure S11 Raman and ATR FTIR 20
Figure S12 ToF SIMS ion distribution of coated glass surface 21
Figure S13 ToF SIMS peak area integration 22
Figure S14 ^{29}Si HPDEC-MAS spectrum 23
Figure S15 EDX of pristine and coated cellulose filter 24
Figure S16 DLS of pristine and coated silica nanoparticles 24
Literature 25

Experimental Section

Synthesis of poly(S_n-r-StyTMS)

500 mg of Sulfur powder (> 99%, Alfa Aesar, Ward Hill, USA) and 500 mg of Styrylethyltrimethoxysilane (Gelest, Morrisville, USA) were weighed into a vial with a stir bar and sealed with a cap. The vial was heated to 130 °C for 8 h under stirring at 400 rpm. The vial was extracted stepwise with 4, 2 and 2 mL of THF, respectively. The solution was cooled to - 20 °C for 20 min to precipitate residual and excess sulfur, which was then removed by centrifugation. Evaporation of volatiles yielded a THF soluble, brown oil. For the experiments, poly(S_n-*r*-StyTMS) was not isolated but instead used directly in THF solution after isolation from sulfur.

Solution processing of poly(S_n-r-StyTMS)

Poly(S_n-*r*-StyTMS) was diluted with THF to reach a mass concentration of 40 mg mL^{-1} (rel. to the initial mass of styrylethyltrimethoxysilane). Poly(S_n-*r*-StyTMS) was hydrolyzed by stirring with 5 vol% of dilute HCl (pH 4) for 60 min at room temperature.

Spin coating of silicon surfaces for ellipsometry

Ca. 250 µL of hydrolyzed poly(S_n-*r*-StyTMS) were pipetted onto pieces (ca. 2 × 2 cm) cut from a silicon waver until it was fully covered with liquid. The waver pieces were then spun at 6000 rpm for 35 s.

Spin coating of surfaces for AFM scratch analysis

All substrates were spun covered in poly(S_n-*r*-StyTMS) solution at 1500 rpm for 15 s. The same volume was then added on the substrate within 10 s and it was spun at 1500 rpm for 15 s again. For silicon, pieces cut from a silicon waver (ca. 2 × 2 cm) 250 µL were used for both pipetting steps. For gold coated glass slides and glass slides (2.5 × 7.5 cm) ca. 800 µL of hydrolyzed poly(S_n-*r*-styrylsilane) were used for both pipetting steps. For consecutive spinning cycles the specimen were air dried for at least 20 min before the next spinning cycle. Glass slides were activated by immersion into fuming HCl:MeOH (1:1 V:V) for at least one day. The water contact angles before and after activation were 49.4° ± 0.2° and 31.3° ± 0.3°, respectively.

Synthesis of net-poly(S_n-r-StyTMS) coated silica particles

3.00 g of sulfur powder (> 99%, Alfa Aesar, Ward Hill, USA) and 3.00 g of styrylethyltrimethoxysilane (Gelest, Morrisville, USA) were weighed into a vial with a stir bar and sealed with a stopper. The vial was heated to 130 °C for 8 h under stirring at 400 rpm. The vial was extracted with THF and poured into a beaker. The solution was then decanted to remove residual sulfur and filled up to a total of 300 mL THF. 50.13 g Silica particles (40-63 µm) were charged into a 1 L round bottom flask and the poly(S_n-*r*-

StyTMS) solution was poured in. 1.5 mL of H_2O (pH 4) were added and the dispersion was stirred vigorously for 24 h. The mixure was then filtered through a buchner filter. The filter cake was briefly (10 min) dried in an oven at 80 °C. The particles were then washed with 80, 50, and 3 × 20 mL of THF and then with 3 × 20 mL of CS_2, until the washings were colorless. They were then dried for 20 min at 110 °C. A total of 51.86 g coated particles could be obtained.

Synthesis of net-poly(S_n-r-StyTMS)

Hydrolyzed poly(S_n-*r*-StyTMS) was charged in a Teflon mold or round bottom flask. Volatiles were removed at room temperature, in an oven, or in a rotary evaporator. The yielded orange-red solid was washed thoroughly with THF and CS_2. For DSC, TGA, solid state NMR and PXRD measurements, the orange-red solid was milled into a powder and was allowed to age for two months.

Mercury Removal Test

Silica particles coated with *net*-poly(S_n-*r*-StyTMS) (200 mg) were stirred in 10 mL of aqueous $HgCl_2$ solution (15 mg L^{-1}) for 1 h. The dispersion was then centrifuged, and ca. 3 mL of the solution were decanted and filtered. The filtrate was diluted with 1:30 v/v% water (HPLC grade) and 300 µL of concentrated HCl were added. The Hg^{2+} concentration was determined with Hydride-AAS (atom absorbance spectroscopy). Pristine silica particles (40 – 63 µm) were used as a reference. The pH of the filtrate after the remediation experiment was neutral.

Thin film interference color calculation

To calculate the thin film interference in dependence of viewing angle and thickness, a script created by Dr. Jens Raacke was used (http://www.raacke.de/index.html?airy.html).

Based on the Cauchy parameters n_0, n_1 and n_2 and the absorption coefficients k_0, k_1 and k_2 the functions n(λ) and k(λ) are calculated in steps of 5 nm from 380 nm to 780 nm. n and k are combined as complex refractive index N according to the Kramers-Kronig relation (I) for further calculations.

(I) $$N = n + ik$$

The following calculations (1 and 2) are then executed for every data point, i.e. for all linear combinations of viewing angle and thickness from 20 - 40° and 0 - 300 nm, respectively.

1) Calculation of the reflection and transmission coefficients of the transverse electric (TE) and transverse magnetic (TM) polarizations based on the Fresnel law. $E_{0,i}$, $E_{0,t}$ and $E_{0,r}$ are the electric field strength of the incoming, transmitted and reflected beam, respectively.

(II) $$t_{TE} = \left(\frac{E_{0,t}}{E_{0,i}}\right)_{TE} \qquad t_{TM} = \left(\frac{E_{0,t}}{E_{0,i}}\right)_{TM}$$

(III) $r_{TE} = \left(\frac{E_{0,r}}{E_{0,i}}\right)_{TE}$ $r_{TM} = \left(\frac{E_{0,r}}{E_{0,i}}\right)_{TM}$

2) The refracted beams are a superposition of multiple beams. To calculate the total reflectivity, the sum of all refracted beams is formed based on series development.

3) The reflectivity spectrum is multiplied with the sensitivity spectra of all three human color receptors to obtain a XYZ color scheme.

4) The obtained XYZ colors are converted to RGB colors.

5) Light source correction from Illuminant E (emission intensity assumed constant for all wavelengths of the visible light spectrum) to the selected light source (5300 K) and averaging over TE and TM polarization.

Methods

Specifications of analytical devices in an alphabetical order are listed below.

AFM

The surface topology of samples was investigated with a *Dimension Icon* with *ScanAsyst* from Bruker (Billerica, USA). Cantilevers with a resonance frequency of 325 kHz from Olympus (Shinjuku, Japan) were used.

ATR FTIR

Powdered *net*-poly(S_n-*r*-StyTMS) was investigated with a *Bruker Tensor 27* ATR FTIR spectrometer from *Bruker Optik* (Ettlingen, Germany) in 45° geometry on a diamond crystal (single reflection).

DLS

Nanosized particles were measured on a *Zetasizer Nano ZS* from Malvern Instruments (Malvern, United Kingdom) equipped with a 633 nm laser.

DSC

Differential Scanning Calorimetry was performed on a *Discovery DSC* from *TA Instruments* (Newcastle, USA). The heating rate was 10 K min^{-1}.

EDX/SEM

Specimen were analyzed with a *LEO 1530* scanning electron microscope from *Leica* (Hillsboro, USA) with an accelerating voltage of 5-10 kV. For SEM analysis the specimen were sputtered with a thin layer of gold. For EDX a *NORAN System SIX* from Thermo Scientific (Waltham, USA) was used.

Ellipsometry

Spectroscopic Ellipsometry measurements were performed on a *M2000* from Woollam (Lincoln, USA). Samples were prepared via spin-coating on silicon wafers (ca. 2 × 2 cm^2). Measurements were taken at an angle of incidence of 45°, 55° and 65° in the spectral range of 300-1000 nm. To evaluate the experimental data, an optical box model was applied using the instrument software *CompleteEase* (V6.51). Silicon substrates were fitted with database values for Si and SiO_2.[1] To determine the thickness and optical constants of the polymer layer, all spectra of samples of varying thickness were fit together in a multi-sample analysis. For this, the Cauchy function with Urbach extension terms was used in order to consider the absorption band of the polymer in the UV range. Common fit parameters were A_n, B_n, as well as A_k and B_k. The thickness was allowed to vary freely between samples. As the band edge, 400 nm was assumed.

GPC

Samples were dissolved in THF (ca. 2 mg mL^{-1}), filtered through 0.43 µm PTFE filters and injected in a *Tosoh EcoSEC* GPC system from *Tosoh* (Tokio, Japan) equipped with a SDV 5 µm bead size guard column (50 × 8 mm) followed by three SDV 5 µm columns (300 × 7.5 mm, subsequently 100, 1000, and 105 Å pore size, PSS), a differential refractive index (DRI) detector, and a UV-Vis detector set to 254 nm. THF was used as eluent at 35 °C with a flow rate of 1.0 mL·min^{-1}. The SEC system was calibrated by using linear polystyrene standards ranging from 800 to 1.82 × 10^6 g mol^{-1}.

NMR

^{1}H NMR spectra were recorded on a *Bruker Avance III 400 MHz* spectrometer from *Bruker* (Rheinstetten, Germany) using $CDCl_3$ (99.8 atom% D, 1 v/v% TMS) from *Merck* (Darmstadt, Germany) as a solvent. For one spectrum 128 scans were collected. ^{29}Si NMR experiments were performed on a *Bruker Avance Neo 400 NB* spectrometer equipped with a 5-mm BBO probe from *Bruker* (Rheinstetten, Germany) at a read-out temperature of 298 K. The spectra of styrylethyltrimethoxysilane and poly(S_n-*r*-StyTMS) were recorded with 512 and 2560 scans, respectively, and a recycle delay of 60 s was used. Samples were dissolved in $CDCl_3$ (99.8% D, 0.03 v/v% TMS) from ARMAR *AG* (Döttingen, Switzerland). The optimized ^{29}Si 90° pulse length was 10.5 µs. WALTZ-16 heteronuclear decoupling was used during the acquisition. ^{29}Si chemical shifts were externally referenced to the ^{29}Si signal of TMS at 0 ppm.[2] Processed data were further analyzed using MestReNova 14.1.0 from *Mestrelab Research S.L.* (Santiago de Compostela, Spain).

Solid-state NMR

^{29}Si high-power decoupled (HPDEC) and cross-polarization (CP) magic-angle spinning (MAS) NMR spectra were recorded using a *Bruker Avance III 400 WB* spectrometer from *Bruker* (Rheinstetten, Germany) equipped with a 4-mm double resonance MAS probe at a read-out temperature of 300 K. The spectra were acquired at a spinning speed of 12 kHz using TMS at 0 ppm as external reference. Optimized ^{1}H and ^{29}Si 90° pulse lengths were 2.5 and 5.97 µs, respectively. The ^{29}Si CP spectrum was acquired with 1024 scans and a recycle delay of 5 s. ^{1}H to ^{29}Si magnetization transfer was achieved by using linear 70–100% ^{1}H-ramped CP with a contact time of 8 ms to fulfill the Hartmann–Hahn condition.[3] Heteronuclear decoupling during acquisition was achieved with swept-frequency two-pulse phase modulation (SW_f-TPPM).[4,5] For HPDEC experiments, 664 and 736 scans were collected for powdered *net*-poly(S_n-*r*-StyTMS) and the background, respectively, with a recycle delay of 90 s. SW_f-TPPM

heteronuclear decoupling was used during the acquisition. Processed data were analyzed using *MestReNova 14.1.0* from *Mestrelab Research S.L.* (Santiago de Compostela, Spain).

PXRD

Powder X-ray diffraction was measured using a *PANanlytical X´Pert PRO* diffractometer from *Malvern Panalytical* (Malvern, UK), operating in transmission geometry. All data were measured over the range 5-50° 2θ.

Raman

Raman spectra were recorded on a *MultiRAM* from *Bruker Optik* (Ettlingen, Germany) equipped with a Nd:YAG (1064 nm) laser.

UV/VIS

Spectra were recorded with a *PerkinElmer Lambda 35* UV/VIS spectrometer from *PerkinElmer Ink.* (Waltham, USA). Solutions were measured in quartz cuvettes against a background of HPLC grade water.

TGA-MS

Powdered *net*-poly(S_n-*r*-StyTMS) was measured on a *STA 449 C Jupiter* with a sample holder from *Netzsch-Gerätebau GmbH* (Selb, Germany) equipped with a quadrupole mass spectrometer *403 C Aëolos* from *InProcess Instruments* (Bremen, Germany). The measurement conditions applied were as follows: heating rate 10 K min^{-1}, temperature range 50–400 °C, streaming synthetic air (SynA: 79 mass% N_2, 21 mass% O_2; 50 mL min^{-1}) as purge gas and 20 mL min^{-1} N_2 as protective gas. An empty Pt/Rh crucible (diameter 5 mm, height 5 mm) with a punched lid was used as inert reference sample. The gas released was detected by mass spectrometry. The following mass fragment numbers m/z were investigated: = 18, 44, 64.

ToF SIMS

ToF-SIMS (time-of-flight secondary ion mass spectrometry) was performed on a *TOF.SIMS5* from *ION-TOF GmbH* (Münster, Germany), equipped with a Bi cluster liquid metal primary ion source and a non-linear time-of-flight analyzer. A surface area of 0.25 mm² (500 × 500 µm) was investigated. The Bi source was operated in bunched mode providing 0.7 ns Bi^{1+} ion pulses at 25 keV energy and a lateral resolution of approx. 4 µm. The short pulse length allowed for high mass resolution to analyze the complex mass spectra of the immobilized organic layers.

XPS

XPS spectra were recorded on an *Axis Ultra DLD* from Shimadzu (Kyoto, Japan) utilizing monochromatized Al Kα radiation. The survey scan and the high-resolution scans were operated at an analyzer pass energy of 160.0 eV and 40.0 eV, respectively. Recorded spectra were calibrated on the S $2p_{3/2}$ peak at a binding energy of 164.0 eV.

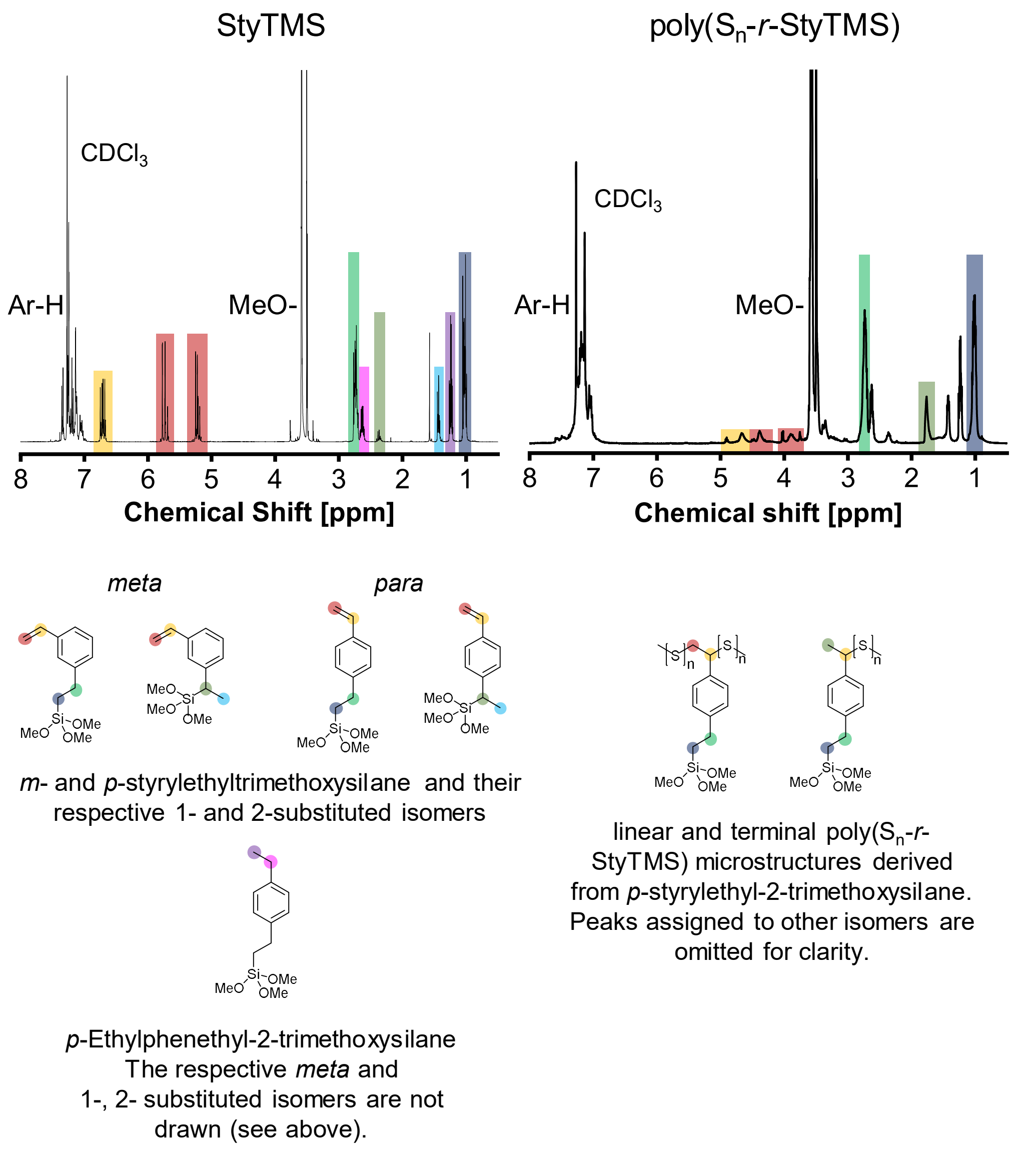

Figure S1. ^{1}H NMR spectrum of styrylethyltrimethoxysilane (StyTMS) and the product of the inverse vulcanization reaction of StyTMS with elemental sulfur, poly(S$_n$-*r*-StyTMS). StyTMS is a mixture of the *para* and *meta* isomer as well as the respective 1- and 2-substituted products of the hydrosilylation reaction of divinylbenzene (mixture of *meta* and *para*) and trichlorosilane. An impurity in commercial divinylbenzene is (*m*, *p*)-ethylstyrene. Due to the mixture of isomers the spectrum was color coded for an easier understanding. Based on the integral ratio between the signals at 2.77-2.70 and 2.40-2.34 ppm, the ratio between 2- and 1-substituted styrylethyltrimethoxysilane is ca. 7:1. The relative amounts of the *meta* and *para* isomers cannot be resolved with this spectrum but is presumably similar to the distribution for commercial divinylbenzene (ca. 2:1). The amount of unreactive ethylphenethyl-trimethoxysilane was calculated as 27% based on the integral ratio of the doublet of quartets (2.62 ppm,

Ar-**CH_2**-CH_3) of ethylphenethyltrimethoxysilane relative to the multiplet (2.71 ppm, Si-CH_2-**CH_2**-Ar) shared by all 2-substitution products. This finding contradicted the manufacturer value (< 10%).

Peaks corresponding to isomers of styrylethyltrimethoxysilane were assigned as follows:

^{1}H NMR (400 MHz, CDCl3) δ = 7.35-7.02 (4H, m, Ar-H), 6.75-6.68 (1H, m, Ar-C**H**=CH_2), 5.77-5.73 (1H, d, *cis*-vinyl), 5.25-5.22 (1H, d, *trans*-vinyl H), 3.58 (9H, s, Si-OMe), 2.77-2.70 (2H, m, Ar-C**H_2**-CH_2-Si), 2.40-2.34 (1H, m, Ar-C**H**-(CH_3)Si), 1.45-1.41 (3H, m, Ar-CH(Si)-C**H_3**), 1.05-1.00 (2H, m, Ar-CH_2-C**H_2**-Si).

Impurities: MeOH (3.49 ppm), H_2O (1.56 ppm), ethylphenethyltrimethoxysilane (2.67-2.60, 2H, m, Ar-C**H_2**-CH_3 and 1.26-1.21, m, Ar-CH_2-C**H_3**).

The spectrum of poly(S_n-*r*-StyTMS) showed quantitative conversion of vinyl groups, thus all four isomers (*meta*, *para* and the respective 1- and 2-substituted isomers) are reactive in the inverse vulcanization reaction. The ^{29}Si satellites of the methoxy peak at 3.58 ppm were retained in the poly(S_n-*r*-StyTMS) spectrum, indicating that the methoxy groups were still connected to the silicon center after the inverse vulcanization reaction. The emergence of peaks at 4.67, 4.39, and 3.90 ppm showed formation of C-S bonds. The peak at 1.75 indicated formation of a terminal -CH_3 microstructure as previously observed for styrene.[6]

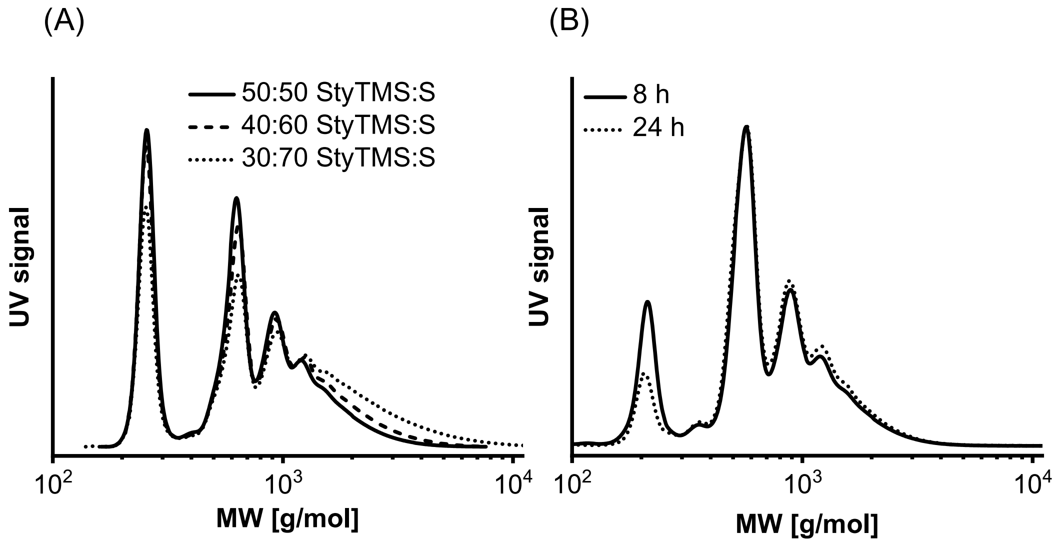

Figure S2. GPC traces of the crude product of the inverse vulcanization reaction (Scheme 1): poly(S_n-*r*-StyTMS). Influence of the feed ratio (A) and the reaction time (B) on the molecular weight (MW) of the product. In (A) use of higher amounts of sulfur led to slightly higher molecular weights (reaction time 8 h). Despite this finding, the sulfur weight content of samples aged for two months did not exceed 35 wt% as determined with elemental analysis. In (B) the weight ratio was 50:50 sulfur to StyTMS. The low molecular weight trace is attributed to ethylphenethyltrimethoxysilane.

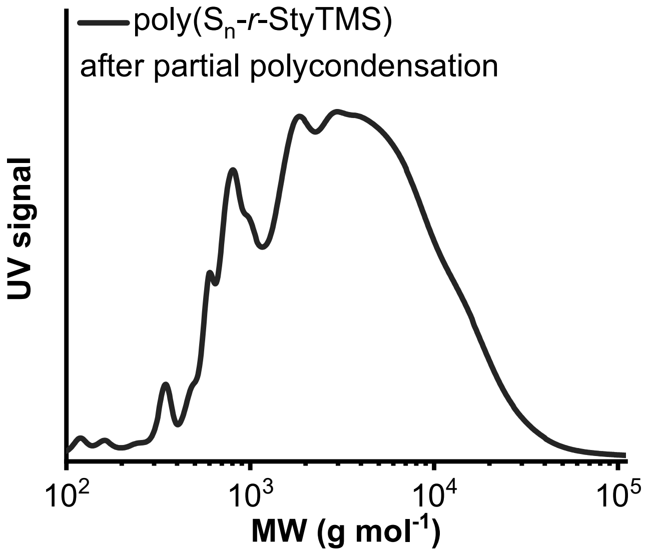

Figure S3. GPC traces of hydrolyzed poly(S_n-*r*-StyTMS) dissolved in THF after three months in a tightly sealed vial. A weight ratio of 50:50 sulfur to StyTMS was reacted for 8h and the product was dissolved in THF. The vial (10 mL) was opened after three months and THF could evaporate to ca. ½ of the initial volume. Phase separation of the former clear orange solution into a red oil and a transparent THF phase occurred. The molecular weight was determined with GPC (M_N = 1.600 D, M_W = 5.500 D).

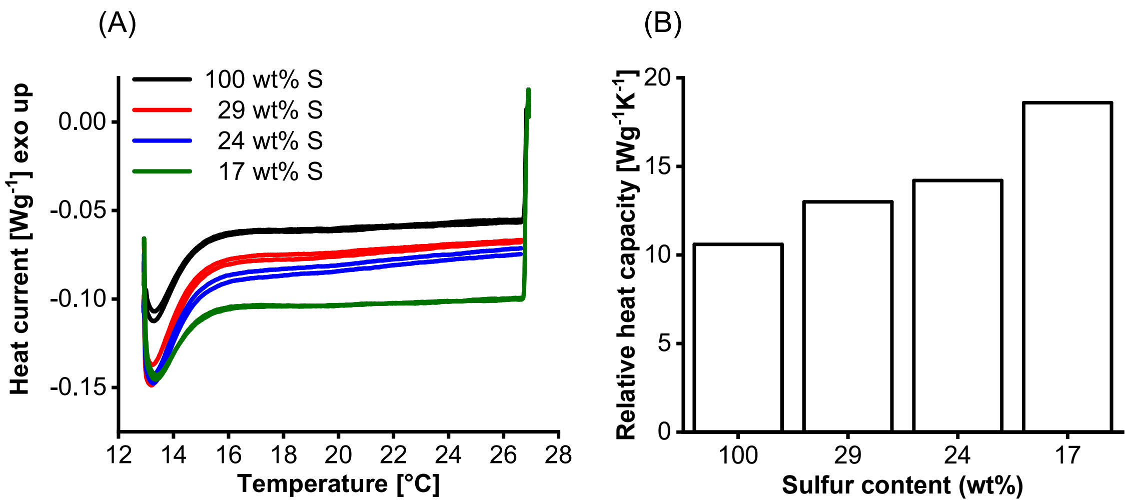

Figure S4. (A) DSC measurements of sulfur powder and *net*-poly(S_n-*r*-StyTMS) powder with different sulfur contents. Each sample was scanned twice from 13 °C to 27 °C at a scan rate of 2 K g^{-1}. (B) Heat capacity [J K^{-1} g^{-1}] of the samples relative to the reference (empty aluminum pan). The heat capacity was calculated for the intervall 18-24 °C. A linear fit of the DSC curves between 16-26 °C was integrated between 18-24 °C. The integrated heat current [W] was then transformed into an energy [J] via the scan rate and then normalized in regards of the temperature. The heat capacity of the formed polymer tended to decrease with increasing sulfur content

To demonstrate the stability of *net*-poly(S_n-*r*-StyTMS) against depolymerization into elemental sulfur, a sample was milled into a powder and washed with THF and CS_2. PXRD (powder x-ray diffractometry) and DSC (differential scanning calorimetry) measurements of powders aged for two months confirmed that no crystalline sulfur had formed (**Figure S4**). This could be concluded from the absence of a sulfur melting peak (DSC) or sulfur crystal reflexes (PXRD). Crosslinked *net*-poly(S_n-*r*-StyTMS) was stable until ca. 220 °C in the presence of oxygen, where it began to decompose (Figure S4 C). Decomposition products above 220 °C were identified to be H_2O, CO_2, and SO_2 by a TGA coupled mass spectrometer. Non-destructive DSC measurements below the degradation zone led to the same result, i.e. no sulfur melting peak was observed (Figure S4 D).

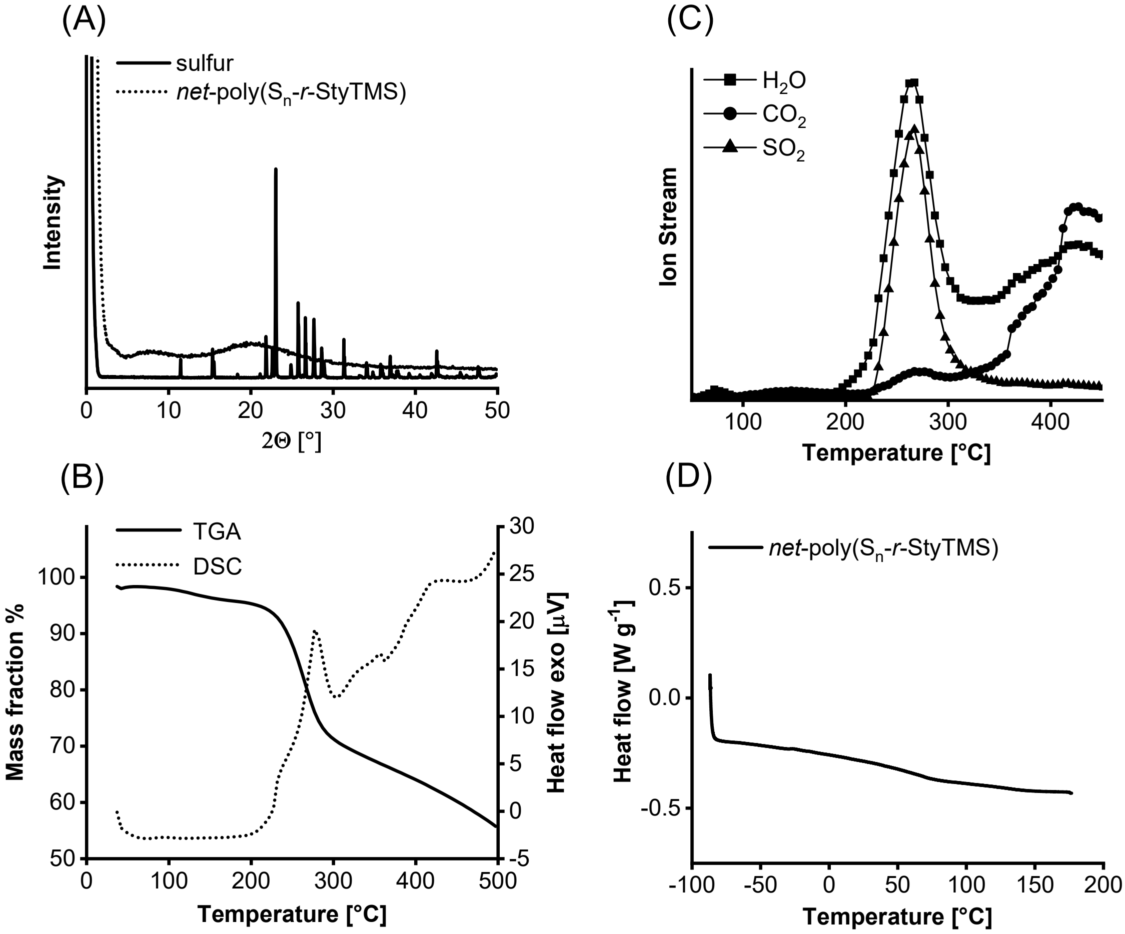

Figure S4. (A) PXRD (powder x-ray diffraction) of *net*-poly(S_n-*r*-StyTMS) powder. The material was amorphous and did not contain crystalline sulfur. (B) DSC (differential scanning calorimetry) of *net*-poly(S_n-*r*-StyTMS) powder. There is no sulfur melting peak (115 °C). The TGA curve (thermogravimetric analysis) showed significant mass loss starting around 220 °C, losing ca. 30% of initial mass until 300 °C and then another 15 wt% until 500 °C were reached. (C) Detected mass fragments during the TGA measurement in air. As apparent from the ion stream, the sulfur containing

molecular fragments were degraded at above 220 °C, whereas the organic fragments were decomposed mainly above 350 °C. Water could originate from residual traces from hydrolysis or result from condensation reactions of residual silanols (D) Non-destructive DSC measurement of *net*-poly(S_n-*r*-StyTMS) powder aged for two months. The sample was heated to 175 °C and then cooled down - 85 °C before the measurement.

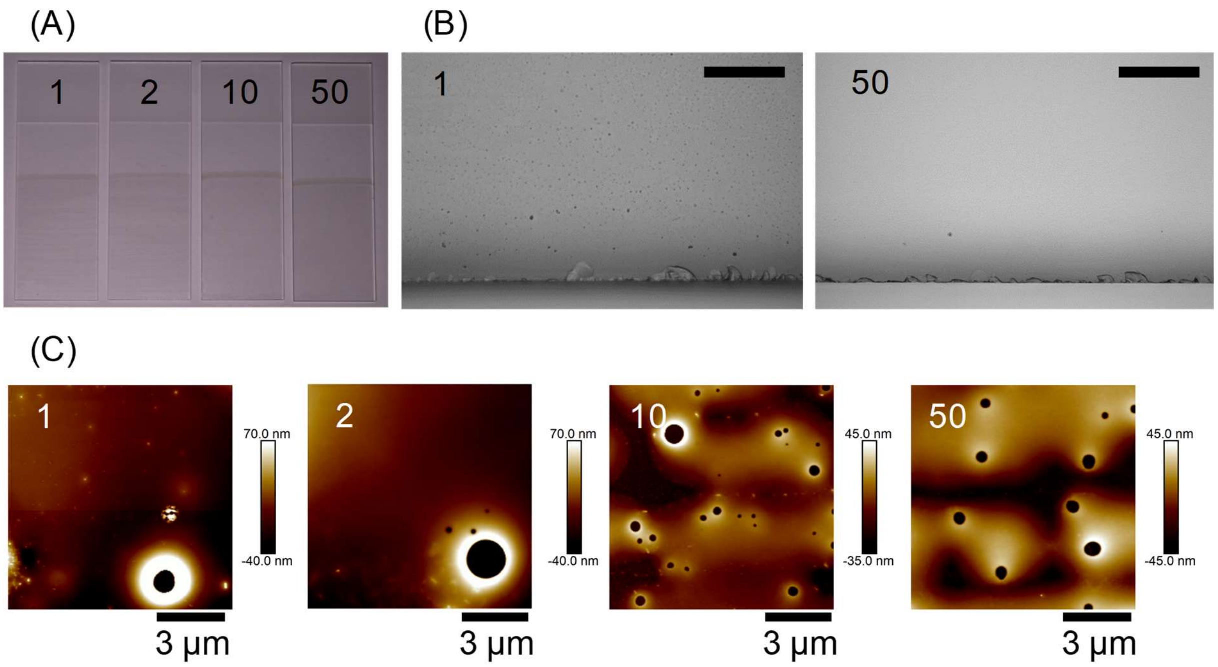

Figure S6. (A) Digital images of dip-coated glass slides using a dip-coater. The numbers are the respective substrate removal speed in device units, with 1, 2, 10, 50 resembling 40, 45, 125, and 760 mm min^{-1}, respectively. (B) Microscopy images at the edges of the glass slides dip-coated with the slowest (“1”) and fastest (“50”) removal speed. While the slow-removal sample appeared to have plenty of micropores on the surface, the fast-removal sample appeared to be smooth. Nonetheless, all samples showed stripes perpendicular to the direction of removal. (C) AFM images of dip-coated glass slides. The heights of the film are ca. 26, 24, 45, and 70 nm for a removal speed of 1, 2, 10, and 50, respectively. The pore size on the features seemed to decrease with increasing removal speed, whereas the number of pores increased.

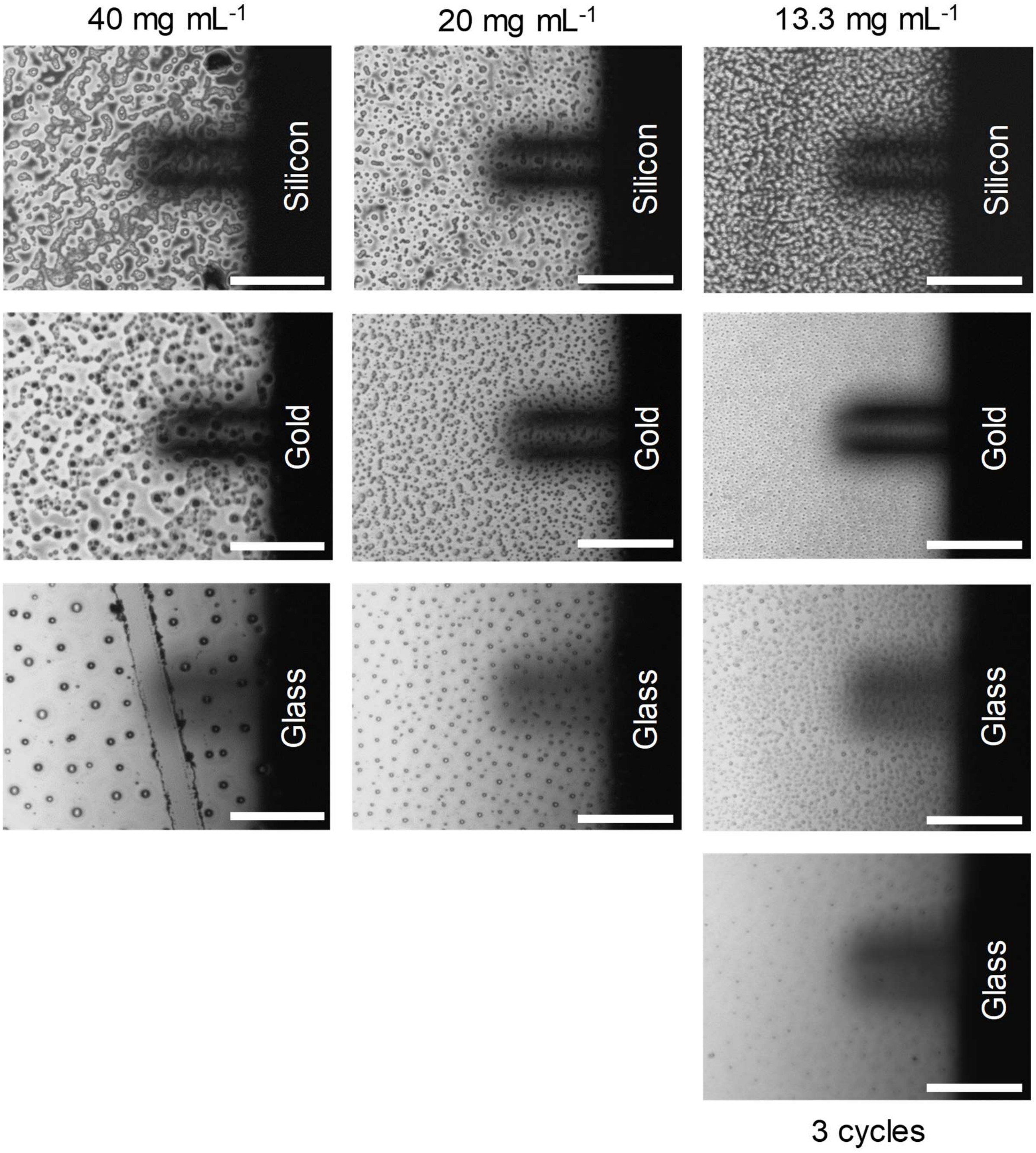

Figure S7. Digital microscope images of surfaces spin-coated with three different concentrations. In the right side of all images the AFM cantilever and tip can be seen. For gold and silicon surfaces periodical features and pores were observed, whose size decreased with decreasing concentration of poly(S_n-*r*-StyTMS). For glass, pores in the surface were observed to decrease in radius with decreasing concentration. Scalebars are 100 μm.

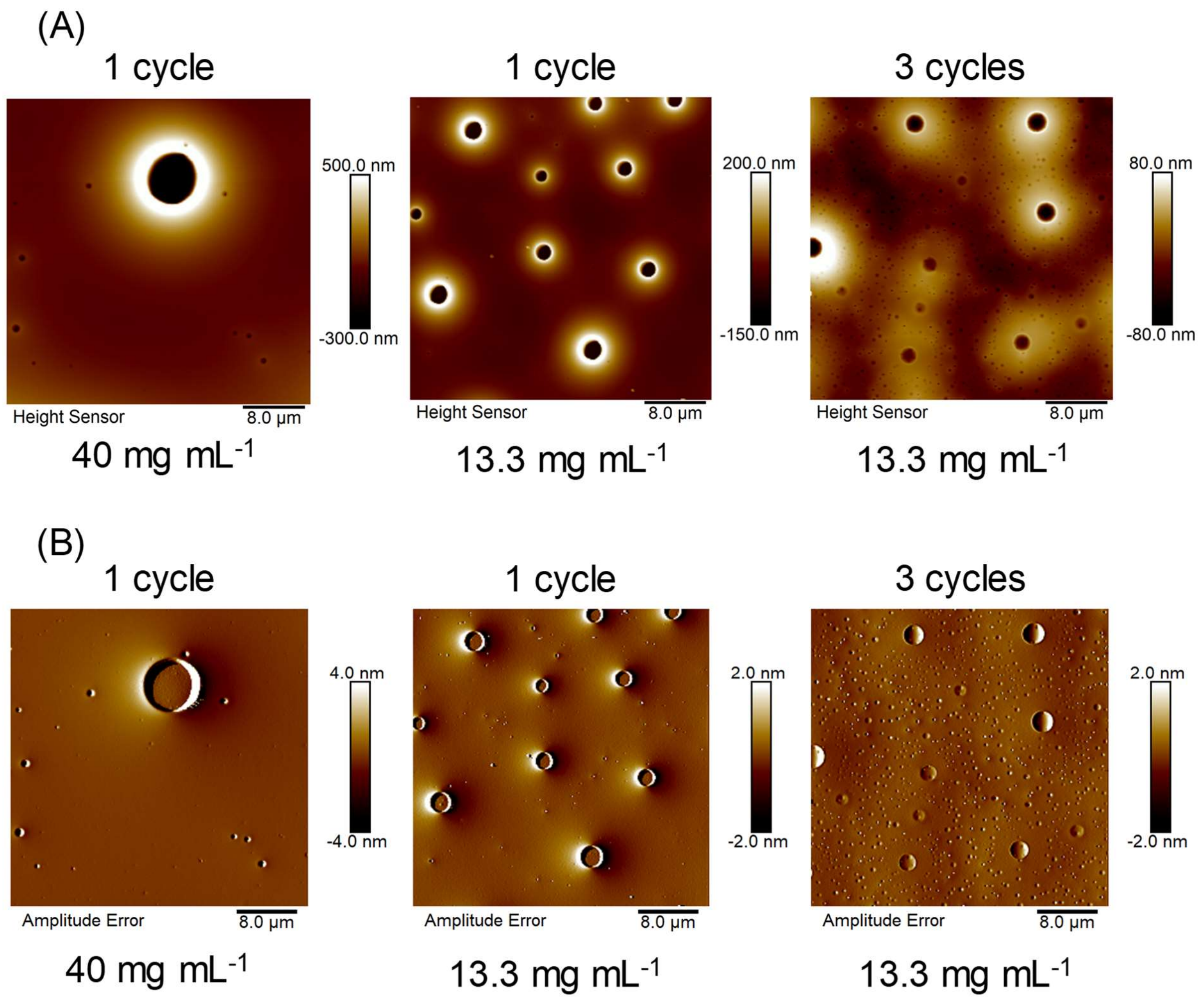

Figure S8. AFM height (A) and amplitude error images (B) of coatings on glass prepared with a concentration of 40 mg mL^{-1} poly(S$_n$-*r*-StyTMS), and with 13.3 mg mL^{-1} for 1 and 3 spin-coating cycles. A shift in the pore size from ca. 6-7 µm (40 mg mL^{-1}) down to ca. 1-2 µm (13.3 mg mL^{-1}) was observed with decreasing concentration. For three spin-coating cycles (13.3 mg mL^{-1}) the formation of nanopores down to ca. 200 nm was observed. The nanopores could be recognized more easily in the amplitude error image. After three times spin-coating the initially formed micropores were partially filled up.

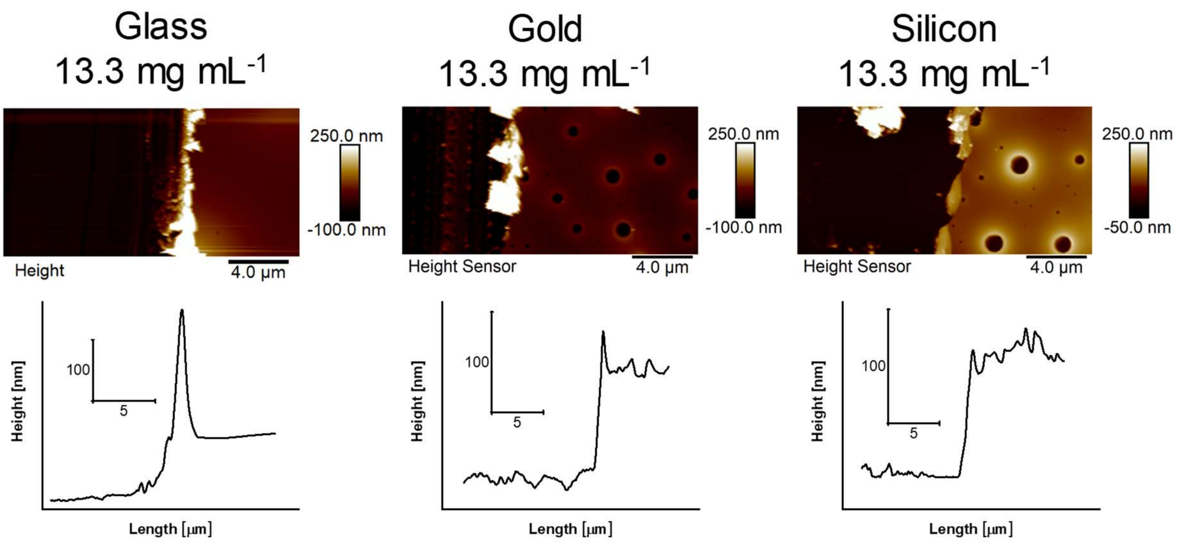

Figure S9. Examples of AFM scratch analysis for surfaces prepared via spin-coating with a concentration of 13.3 mg mL^{-1} poly(S$_n$-*r*-StyTMS) on different substrates. Below are their respective step analysis graphs as analyzed with the Software *NanoScope Analysis 1.5*. For each substrate, three scratches were analyzed to obtain the average and standard deviation. For one scratch a step of at least 3 μm width was averaged to calculate the height difference.

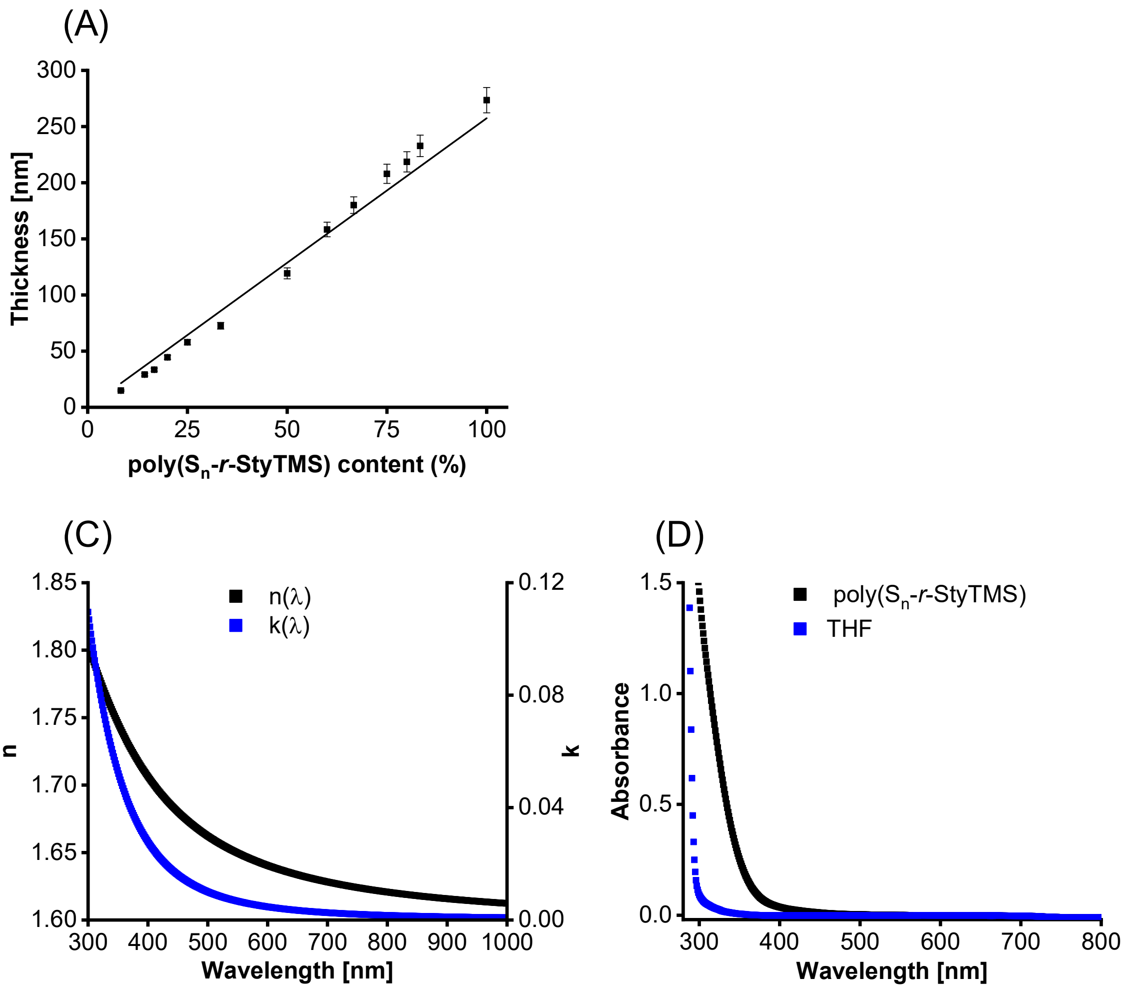

Figure S10. (A) Plot of the *net*-poly(S_n-*r*-StyTMS) film thickness in dependence of the relative poly(S_n-*r*-StyTMS) content with 40 mg mL^{-1} in THF corresponding to 100 %. This solution was diluted with THF to obtain solutions with lower concentrations. Samples were prepared via spin coating (35 s, 6000 rpm) on silicon wafers (2 × 2 cm). Errorbars of thickness values represent the 90% confidence value. The film thickness showed a linear dependence on the relative concentration. Fit result: y = 257.4x (± 6.45), R^2 = 0.992. (B) Plot of the refractive index n and the extinction coefficient k in dependence of the wavelength. Cauchy fit parameters: n = 1.591 + 0.01935 nm^{-2} and k = 0.02425 + 0.1552 nm^{-2}. (D) UV-Vis spectrum of poly(S_n-*r*-StyTMS) in THF confirming the monotonous decrease of light absorbance with increasing wavelength as observed with ellipsometry. Above 550 nm the UV-Vis spectroscope could not detect absorbance relative to the solvent baseline (THF). Sample concentration was 0.16 µg mL^{-1}.

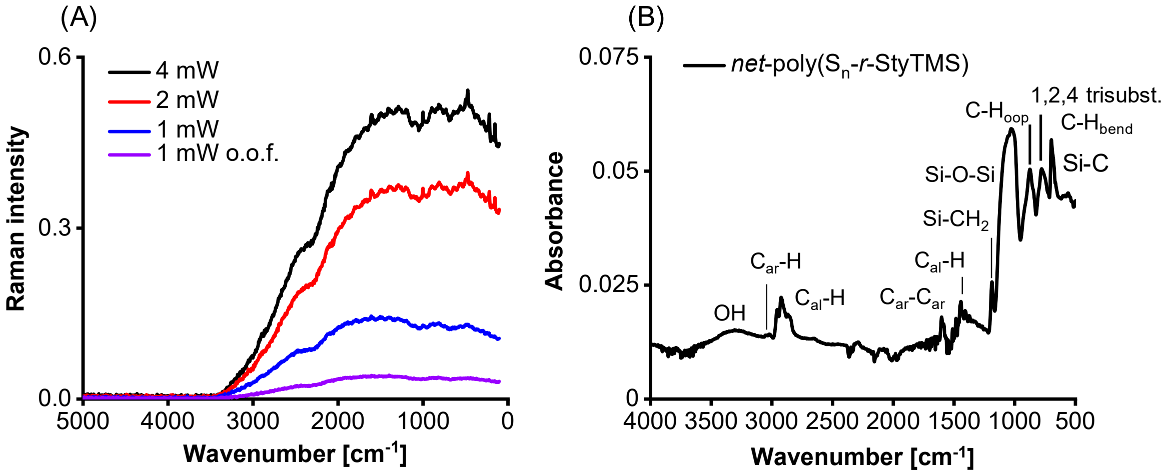

Figure S11. (A) Raman measurements of *net*-poly(S_n-*r*-StyTMS) powder for different laser intensities. With increasing laser energy, we observed an increase in Raman scattering intensity, but were not able to obtain a spectrum due to sample fluorescence. To reduce the laser intensity below the minimum setting of 1 mW, the sample was moved out of focus (o.o.f). (B) ATR FTIR (attenuated total reflection Fourier transform infrared spectroscopy) spectrum of *net*-poly(S_n-*r*-StyTMS). Position v [cm^{-1}] and width of relevant signals: 3300 (mb), 3048 (vw), 3018 (vw), 2957 (vs), 2923 (vs), 2867 (vs), 1602 (s), 1587 (ms), 1512 (ms), 1442 (s), 1188 (vs), 1031 (vs, b), 876 (vs, b), 778 (vs, b), 698 (vs, b).

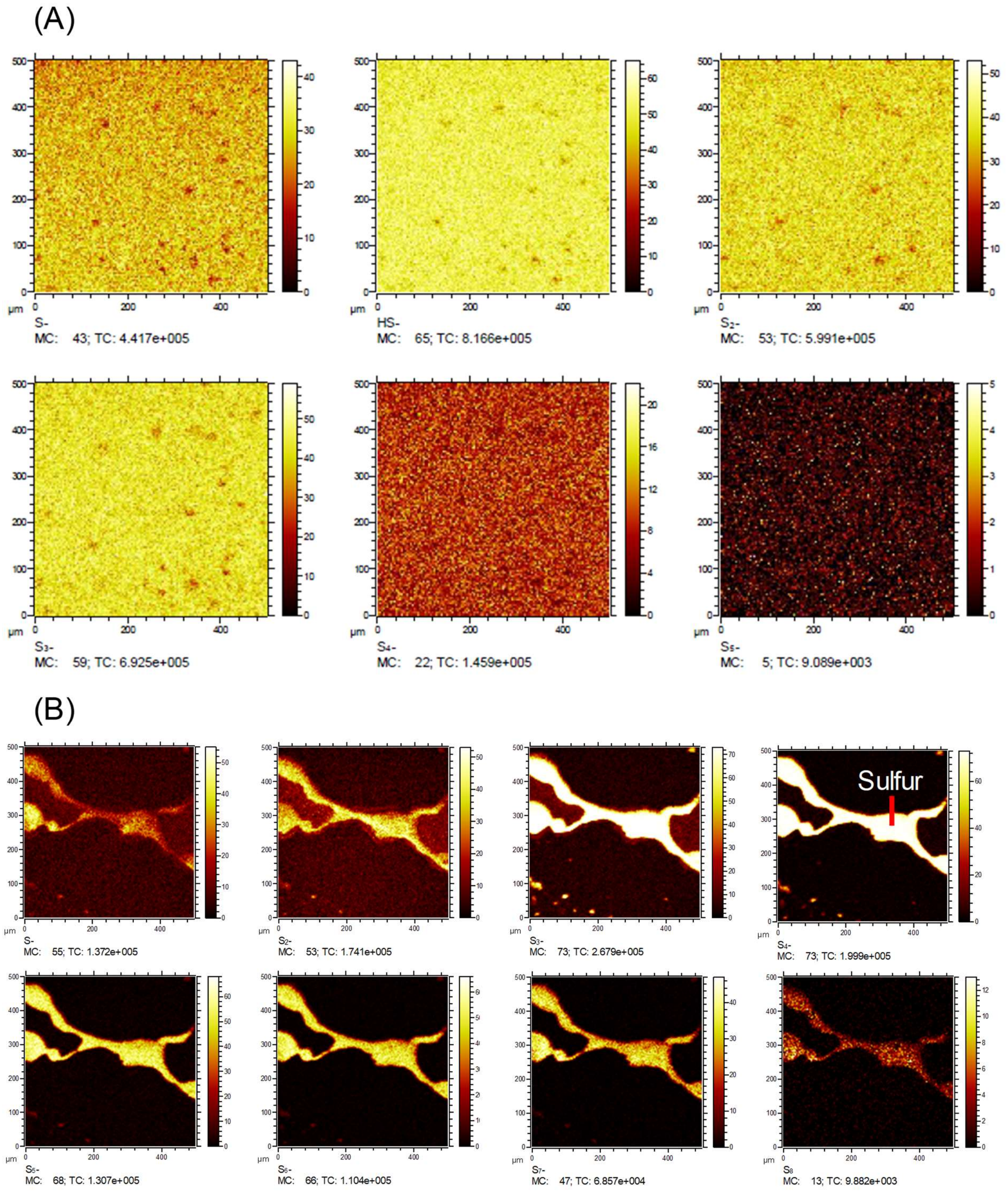

Figure S12. ToF-SIMS images for a surface area of 500 × 500 µm. The spatial distribution of sulfide fragments S_n^- (n = 1-5 or 1-8) was visualized for (A) dip-coated *net*-poly(S_n-*r*-StyTMS) and (B) elemental sulfur spin-coated from CS_2 solution. For *net*-poly(S_n-*r*-StyTMS) no significant amount of fragments higher than S_4^- were detected, while for elemental sulfur fragments up to S_8^- could be observed. Elemental sulfur was not distributed homogenously on the surface due to its fast crystallization upon evaporation of CS_2. Instead, small crystallites formed.

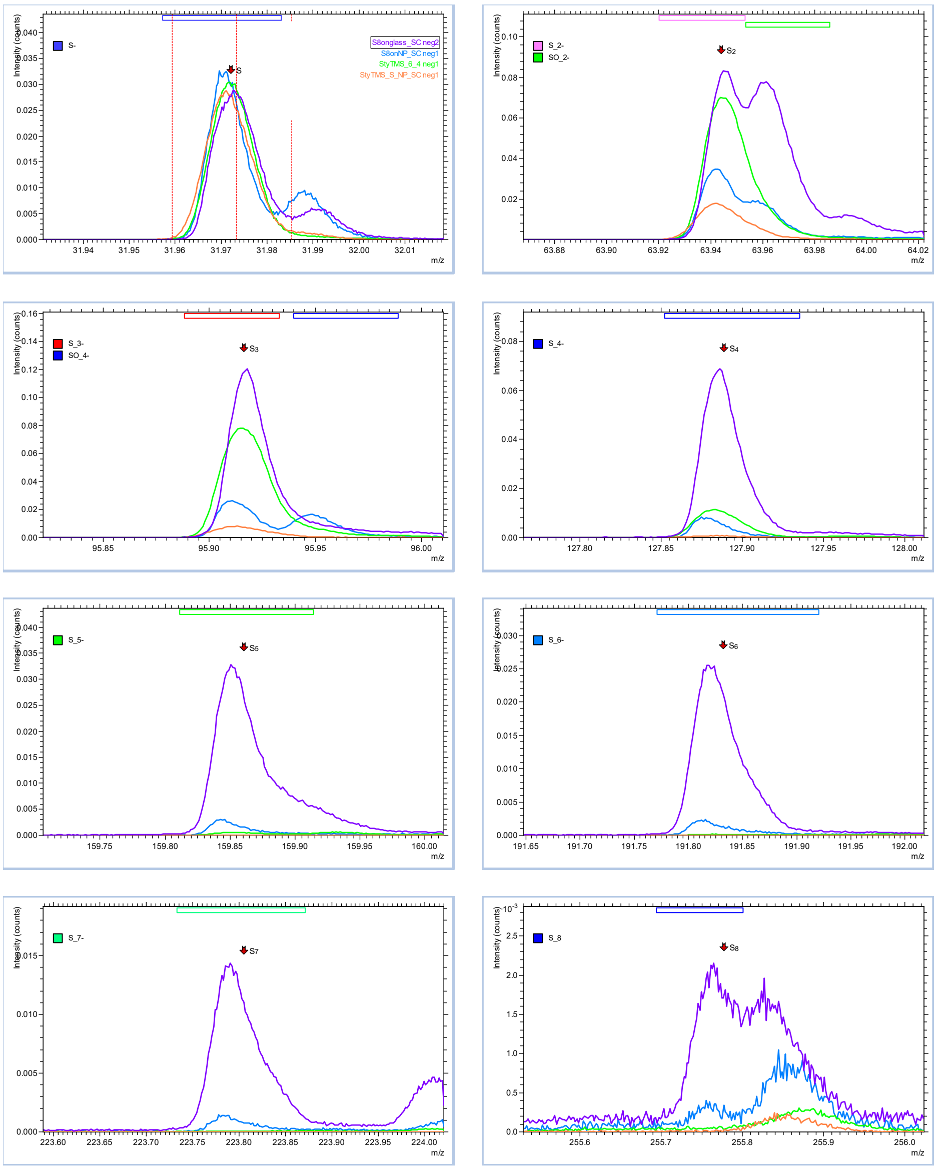

Figure S13. ToF-SIMS peak area integration for S_n^- fragments for n = 1 – 8 for different samples: Elemental sulfur spin-coated from CS_2 on glass (violet) and on silica nanoparticles (blue) as well as *net*-poly(S_n-*r*-StyTMS) coated on glass (green) and on silica nanoparticles (orange). For the comparison in Figure 3D the silica particle free substrates were used. For *net*-poly(S_n-*r*-StyTMS) no significant amount of fragments higher than S_4^- were detected, while for elemental sulfur fragments up to S_8^- were observed.

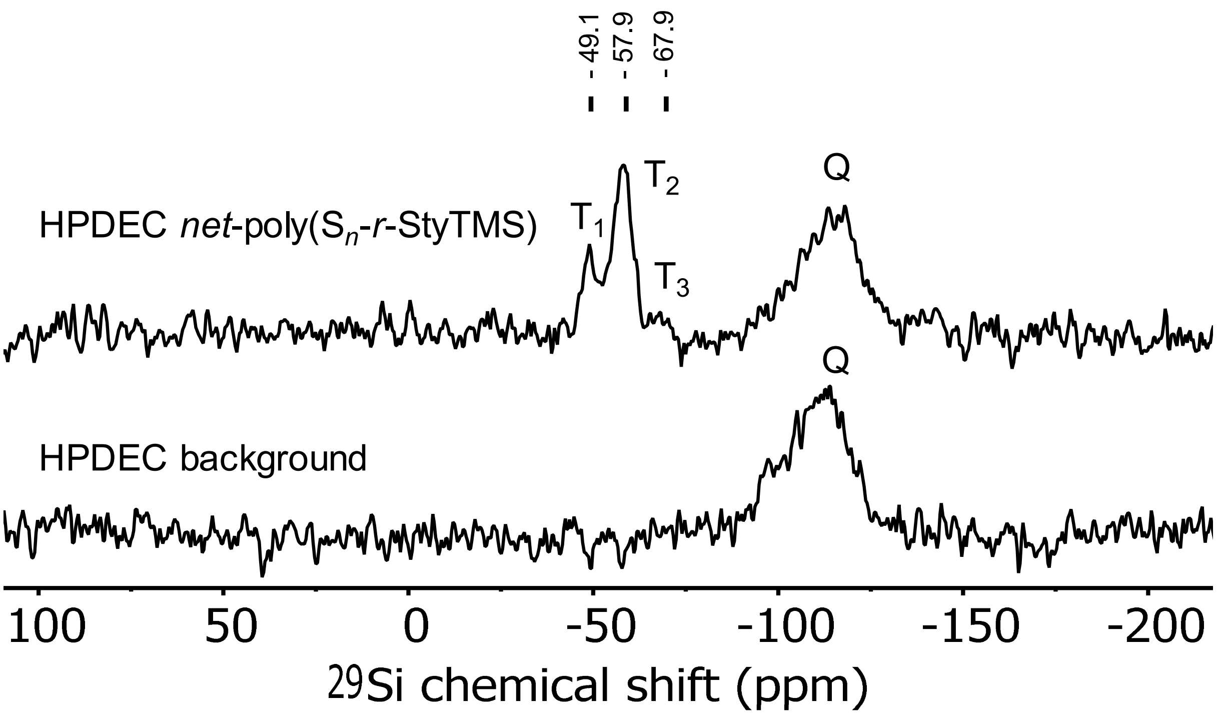

Figure S14. ^{29}Si HPDEC-MAS NMR spectrum of powdered *net*-poly(S_n-*r*-StyTMS) and a background spectrum (rotor). There is an unknown Q species, which is also present in the background control spectrum. Thus, in the sample spectrum only the $T_{1\text{-}3}$ species belong to the sample. Via line fit integration of T_1, T_2, and T_3 an integral ratio of 4.5, 10.6, and 1.0, respectively, was obtained.

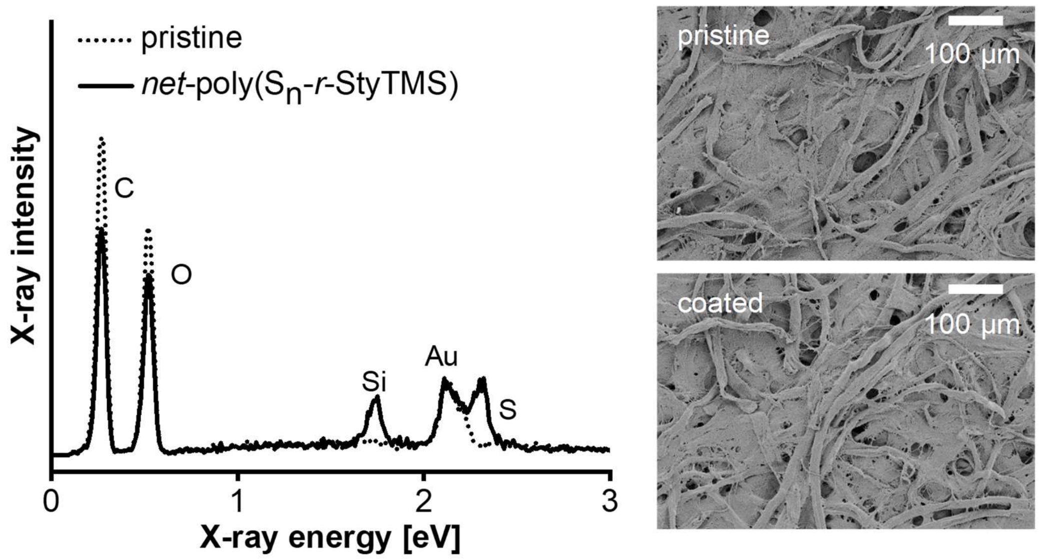

Figure S15. EDX (left) spectrum of a pristine and a *net*-poly(S_n-*r*-StyTMS) coated cellulose filter. The presence of the coating is clearly indicated by the appearance of peaks for sulfur and silicon. SEM images (right) of the cellulose filter before and after coating revealed that the coating does not change the fibrous topology or clog the pores. The surface properties changed from hydrophilic to hydrophobic.

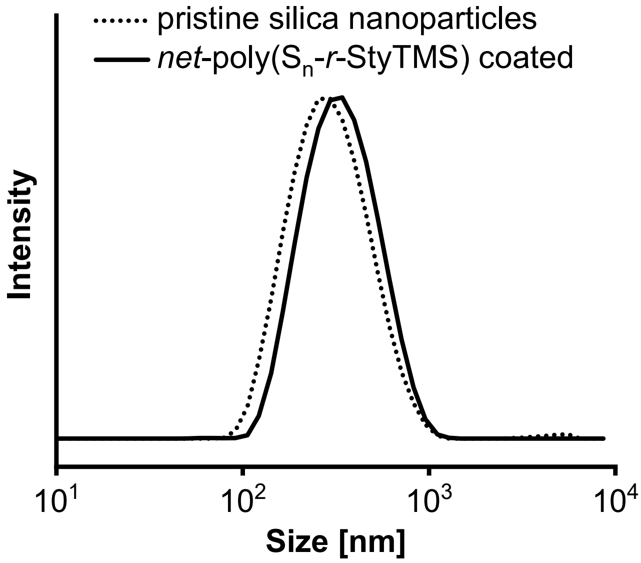

Figure S16. Dynamic light scattering (DLS) measurements of SiO_2 nanoparticles before and after functionalization with *net*-poly(S_n-*r*-StyTMS). After coating with *net*-poly(S_n-*r*-StyTMS) the average particle radius increased from 270 nm to 300 nm. Each curve represents the average of three measurements.

Literature

[1] C. M. Herzinger, B. Johs, W. A. McGahan, J. A. Woollam, W. Paulson, *J. Appl. Phys.* **1998**, *83*, 3323.
[2] Shaka, A., Keeler, J., Freeman, R., *J. Magn. Reson.* **1983**, *53*, 313–340.
[3] Metz, G., Wu, X. L. & Smith, S. O., *J. Magn. Reson. Ser. A* **1994**, *110*, 219–227.
[4] Thakur, R. S., Kurur, N. D., Madhu, P. K. *Chem. Phys. Lett.* **2006**, *426*, 459–463.
[5] Vinod Chandran, C., Madhu, P. K., Kurur, N. D., Bräuniger, T. *Magn. Reson. Chem.* **2006**, *46*, 943–947.
[6] Y. Zhang, J. J. Griebel, P. T. Dirlam, N. A. Nguyen, R. S. Glass, M. E. Mackay, K. Char, J. Pyun, *J. Polym. Sci. A* **2017**, *55*, 107.
